# High frequency of microsatellites in *S. cerevisiae *meiotic recombination hotspots

**DOI:** 10.1186/1471-2164-9-49

**Published:** 2008-01-28

**Authors:** Andrew TM Bagshaw, Joel PW Pitt, Neil J Gemmell

**Affiliations:** 1 School of Biological Sciences, University of Canterbury, New Zealand; 2 Bioprotection and Ecology Division, Lincoln University, New Zealand

## Abstract

**Background:**

Microsatellites are highly abundant in eukaryotic genomes but their function and evolution are not yet well understood. Their elevated mutation rate makes them ideal markers of genetic difference, but high levels of unexplained heterogeneity in mutation rates among microsatellites at different genomic locations need to be elucidated in order to improve the power and accuracy of the many types of study that use them as genetic markers. Recombination could contribute to this heterogeneity, since while replication errors are thought to be the predominant mechanism for microsatellite mutation, meiotic recombination is involved in some mutation events. There is also evidence suggesting that microsatellites could function as recombination signals. The yeast *S. cerevisiae *is a useful model organism with which to further explore the link between microsatellites and recombination, since it is very amenable to genetic study, and meiotic recombination hotspots have been mapped throughout its entire genome.

**Results:**

We examined in detail the relationship between microsatellites and hotspots of meiotic double-strand breaks, the precursors of meiotic recombination, throughout the *S. cerevisiae *genome. We included all tandem repeats with motif length (repeat period) between one and six base pairs. Long, short and two-copy arrays were considered separately. We found that long, mono-, di- and trinucleotide microsatellites are around twice as frequent in hot than non-hot intergenic regions. The associations are weak or absent for repeats with less than six copies, and also for microsatellites with 4–6 base pair motifs, but high-copy arrays with motif length greater than three are relatively very rare throughout the genome. We present evidence that the association between high-copy, short-motif microsatellites and recombination hotspots is not driven by effects on microsatellite distribution of other factors previously linked to both recombination and microsatellites, including transcription, GC-content and transposable elements.

**Conclusion:**

Our findings suggest that a mutation bias relating to recombination hotspots causing repeats to form and grow, and/or regulation of a subset of hotspots by simple sequences, may be significant processes in yeast. Some previous evidence has cast doubt on both of these possibilities, and as a result they have not been explored on a large scale, but the strength of the association we report suggests that they deserve further experimental testing.

## Background

Microsatellites are direct tandem repeats of 1–6 base pair sequence motifs, often strung together in long arrays. They occur much more commonly than expected by chance in the genomes of all eukaryotes [1-3]. The reasons for this are not yet fully understood, but increasing evidence indicates that many microsatellites are functionally important in regulating gene expression [4-10] and possibly also meiotic recombination [11-14]. Microsatellites are also of interest because of their widespread use as genetic markers for applications in genome mapping [15-17], gene hunting [18-20], forensics [21], deducing kinship [22], population genetics [23-25] and the study of the evolution of species [26-28]. These applications depend on assumptions about microsatellite evolution that, at present, are overly simplistic because of unexplained heterogeneity in mutation rates between loci, and an increased understanding of microsatellite evolution and mutational mechanisms is therefore being sought (reviewed in [29,30]).

Slipped strand mispairing during DNA replication is currently thought to cause most microsatellite mutations [31], but it has also been proposed that unequal meiotic recombination could drive microsatellite evolution [32]. Recombination has been demonstrated to cause instability of some microsatellite loci implicated in human disease (reviewed in [33]), but evidence has counted against it being considered a significant factor in microsatellite evolution. Microsatellite instability was not found to be reduced in recombination deficient strains of *E. coli *[34] or *S. cerevisiae *[35] and similar microsatellite mutation rates have been reported for the non-recombining human Y chromosome and the autosomes [36-38]. Also, no association has been found between microsatellite variation and recombination rates on scales of several hundred thousand base pairs in humans [39,40]. Recent evidence has shown, however, that meiotic recombination events predominantly occur in narrow hotspots of 1–2.5 kilo bases (kb) separated by as much as 50–100 kb of DNA that very seldom recombines [41-44]. Data about the relationship between microsatellites and recombination hotspots at this narrow scale are sparse, and there are some signs that it merits further investigation. A poly-AC array inserted near a recombination hotspot in *S. cerevisiae *mutated with high frequency [12], and it has recently been found that polymorphic microsatellites are over-represented in human hotspots [45]. There is also some evidence that microsatellites could have a role in regulating hotspot recombination [11-14], increasing the relevance of studying their association with hotspots, since the basis in sequence of the control of hotspot locations is not yet well understood [42-44,46-48].

It has been shown previously that microsatellite frequencies correlate with broad scale recombination rates in rats, mice and humans [49]. Microsatellites are also associated with intermediate scale recombination rates [50], as well as hotspots in their narrowest known sense [43] in the human genome. So far, however, these studies have reported little detail about the relationship between recombination hotspots and microsatellites. An ideal model organism in which to further examine the association is the yeast *S. cerevisiae*, since it is the simplest eukaryote, and recombination hotspots have been mapped throughout its entire genome [42]. Factors that could complicate an association between microsatellites and recombination are likely to be less problematic in yeast since, for example, the locations of genes and their expression levels have been well-characterized, making it possible to control for the links between microsatellites, recombination and transcription. Also, transposable or other known repetitive elements are not likely to mediate a link between recombination hotspots and microsatellites in yeast, since these elements are not enriched in yeast hotspots [42], as they are in human hotspots [43].

We investigated in detail the association between microsatellites and hotspots of meiotic double-strand breaks (DSBs), the precursors of meiotic recombination, throughout the *S. cerevisiae *genome [42]. As well as long microsatellite arrays, we considered low copy number repeats, which have not been studied previously in relation to recombination, including those with only two copies. This allowed us to address the question of whether recombination is involved in the origin of microsatellites, which has previously been considered to occur mainly by accumulation of random point mutations [51]. An association between low-copy microsatellites and hotspots would suggest the involvement of recombination as a mutational mechanism in microsatellite evolution, since replication slippage is expected to act with significant frequency only on arrays of at least six copies [52-54], and there is no available evidence to suggest that short microsatellites have the potential to stimulate recombination.

We found several types of microsatellite to be strongly associated with recombination hotspots in *S. cerevisiae*, with levels of enrichment greater than two-fold. The associations are, however, stronger for longer microsatellites, and weak or absent for repeats with less than six copies. Our findings suggest that the link between microsatellites and recombination deserves further experimental exploration.

## Results

We used hotspot locations mapped by Gerton and co-workers throughout the *S. cerevisiae *genome using microarray analysis of meiotic DSB frequency [42]. This study identified 177 hotspots, which encompassed all previously known meiotic recombination hotspots in the species, and 40 coldspots. For the purposes of our analysis, we extended the hotspots and coldspots to include the intergenic regions (IGRs) adjacent to the open reading frames (ORFs) identified by Gerton and co-workers [42], since yeast hotspots are typically centred on IGRs, in which most DSBs occur [55]. The hotspots as we defined them have a mean length of 3466 bp. The principal statistical comparisons we made were between hot and non-hot, rather than hot and cold regions, since the cold regions are too few to provide a reliable enough picture of microsatellite density, and recombination frequencies are very low in all experimentally tested regions outside hotspots [41,44].

In general, numbers of repeats are very much lower in ORFs than IGRs, (Table 1), despite the fact that ORFs cover 73.5% of the genome. This is not surprising, since array length change mutations in microsatellites other than tri- or hexanucleotide repeats would cause frame-shifts in ORFs, destroying gene function. Short (3–5 bp) mononucleotide runs have similar frequency in ORFs and IGRs, but this is likely to be due to coding sequence such as AAA (Lys), GTTTTA (Val Leu), GGG (Gly) or AGGGTT (Arg Val), because the vast majority of the short mononucleotide repeats genome-wide are only three bp long. When making comparisons between hot and non hot regions, we accounted for the low microsatellite abundance in ORFs by comparing ORFs exclusively with other ORFs, and IGRs only with other IGRs. We found the abundance of short-motif, AT-rich repeats to be dramatically higher than other repeat types throughout the genome, so we divided microsatellites by motif length as well as by array length in order not to lose information about longer motifs. We also separated poly-A from poly-G. Nineteen physically independent categories of motif and array length were used in total (see Methods section).

**Table 1:** Total number of microsatellite repeats and percentage of regions with at least one repeat in the *S. cerevisiae *genome. The e value denotes the number of bases in any part of a repeat within which no more than one mismatch was allowed with respect to the consensus motif. A lower e value therefore results in the detection of more imperfect repeats.

***Repeat type***			***IGRs***				***ORFs***			
Motif length	Copy number	Mis-matches allowed	Hot (n = 473)		Non hot (n = 5520)		Hot (n = 297)		Non hot (n = 5683)	
			
			No. of repeats	% of IGRs with a rpt.	No. of repeats	% of IGRs with a rpt.	No. of repeats	% of ORFs with a rpt.	No. of repeats	% of ORFs with a rpt

**1 (A)**	6+	perfect	1277	83.1	12547	77.4	339	57.6	13556	74.7
		e = 10	1236	82.2	12262	77.0	338	57.6	13495	74.8
		e = 6	1470	85.4	15153	82.2	437	64.3	17657	80.8
	14+	perfect	79	15.6	409	6.99	4	1.35	30	0.475
		e = 10	146	27.5	741	12.2	5	1.68	73	1.16
		e = 6	173	31.9	917	14.7	7	2.02	132	2.16
**1 (G)**	6+	perfect	33	6.55	241	4.09	32	10.4	474	7.80
		e = 10	32	6.34	240	4.08	32	10.4	474	7.80
		e = 6	46	8.67	307	5.16	44	13.8	641	10.3
	14+	perfect	2	0.423	2	0.0362	0	0	0	0
		e = 10	2	0.423	2	0.0362	0	0	0	0
		e = 6	2	0.423	2	0.0362	0	0	0	0
**2**	6+	perfect	57	10.4	357	6.05	8	2.36	21	0.352
		e = 10	100	18.7	668	11.1	15	4.38	137	2.32
		e = 6	130	23.5	1016	16.3	24	7.07	246	4.12
	10+	perfect	19	3.81	117	2.08	3	1.01	6	0.106
		e = 10	28	5.71	171	3.04	5	1.68	12	0.211
		e = 6	33	6.77	213	3.77	5	1.68	16	0.282
**3**	6+	perfect	7	1.27	27	0.435	8	2.36	165	2.46
		e = 10	11	2.11	66	1.12	20	5.39	316	4.43
		e = 6	21	4.02	118	1.96	28	7.74	478	6.49
	10+	perfect	1	0.211	8	0.145	0	0	29	0.493
		e = 10	3	0.634	17	0.308	0	0	64	1.09
		e = 6	3	0.634	20	0.362	0	0	100	1.57
**4**	6+	perfect	0	0	5	0.0906	0	0	1	0.0176
		e = 10	0	0	12	0.217	0	0	1	0.0176
		e = 6	0	0	19	0.344	0	0	2	0.0352
	10+	perfect	0	0	1	0.0181	0	0	0	0
		e = 10	0	0	1	0.0181	0	0	0	0
		e = 6	0	0	1	0.0181	0	0	0	0
**5**	6+	perfect	0	0	2	0.0362	0	0	0	0
		e = 10	1	0.211	4	0.0725	0	0	3	0.0528
		e = 6	1	0.211	5	0.0906	0	0	4	0.0704
	10+	perfect	0	0	0	0	0	0	0	0
		e = 10	0	0	0	0	0	0	0	0
		e = 6	0	0	0	0	0	0	1	0.0176
**6**	6+	perfect	1	0.211	3	0.0543	0	0	3	0.0528
		e = 10	1	0.211	21	0.326	2	0.673	15	0.246
		e = 6	1	0.211	10	0.181	4	1.35	11	0.176
	10+	perfect	0	0	0	0	0	0	0	0
		e = 10	0	0	9	0.145	1	0.337	1	0.0176
		e = 6	0	0	4	0.0725	1	0.337	5	0.0704

### High microsatellite frequencies in meiotic recombination hotspots

**Figure 1:**
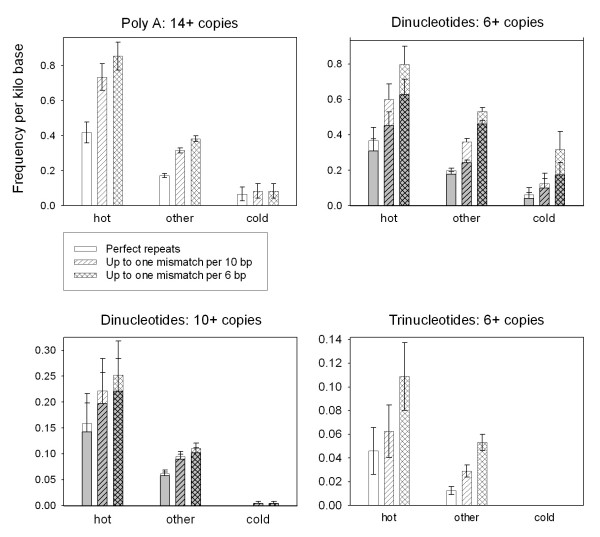
**Frequencies of high-copy, short-motif repeats in yeast intergenic regions**. Mean microsatellite frequencies in *S. cerevisiae *IGRs divided according to DSB intensity into 473 hot, 89 cold and 5431 other regions, which were all IGRs not categorized as either hot or cold. Poly-AT arrays comprised the majority of dinucleotide repeats and are highlighted in grey. Error bars are plus and minus one SEM.

Microsatellite frequencies in meiotic recombination hotspots and non-hot regions of the *S. cerevisiae *genome can be found in Additional file 1, Tables S1 and S2. Several types of microsatellite have significantly different frequency in hot than non-hot areas (alpha, adjusting for Bonferroni's correction = 0.0026, Table 2). Repeat frequencies in the 40 coldspots are generally lower than in other non-hot regions, but these differences are not statistically significant (Additional file 1, Tables S1 and S2). The correlation between DSB intensity level, assayed for all yeast ORFs by Gerton and co-workers [42], and microsatellite frequency, is generally weak (Additional file 1, Tables S3 and S4), but several repeat types, especially long poly-A and dinucleotide microsatellites, are markedly more abundant in hotspots than non-hot regions (Figure 1, Table 2).

**Table 2:** Microsatellite types with a significant difference in frequency either between hot and non-hot IGRs, or hot and non-hot ORFs, in the *S. cerevisiae *genome. Significance was inferred where p < 0.0026, with the level of alpha adjusted for 19 independent classes of repeat using Bonferroni's correction. The Mann-Whitney U Test or T Test was used, depending whether samples were normally distributed. The e value denotes the number of bases in any part of a repeat within which no more than one mismatch was allowed with respect to the consensus motif. A lower e value therefore results in the detection of more imperfect repeats.

***Repeat type***			***IGRs***				***ORFs***			
Motif length	Copy number	Mis-matches allowed	Mean per kb freq.		Freq. ratio (hot/non hot)	P value	Mean per kb freq.		Freq. ratio (hot/non hot)	P value
								
			Hot	Non hot			Hot	Non hot		

**1 (A)**	3 to 5	perfect	35.0	39.9	0.88	< 0.0001	29.2	36.1	0.81	< 0.0001
		e = 10	34.3	39.4	0.87	< 0.0001	29.1	36.0	0.81	< 0.0001
		e = 6	31.8	36.7	0.87	< 0.0001	28.0	34.7	0.81	< 0.0001
	6+	perfect	5.42	4.61	1.17	< 0.0001	0.981	1.64	0.60	< 0.0001
		e = 10	5.24	4.50	1.16	< 0.0001	0.978	1.64	0.60	< 0.0001
		e = 6	6.12	5.53	1.11	0.00173	1.28	2.13	0.60	< 0.0001
	14+	perfect	0.418	0.171	2.45	< 0.0001	0.0134	0.00733	1.83	n/s
		e = 10	0.733	0.311	2.36	< 0.0001	0.0182	0.0166	1.10	n/s
		e = 6	0.854	0.377	2.26	< 0.0001	0.0218	0.0271	0.80	n/s
**1 (G)**	3 to 5	perfect	9.18	7.25	1.27	< 0.0001	12.9	10.1	1.28	< 0.0001
		e = 10	9.16	7.24	1.27	< 0.0001	12.9	10.1	1.28	< 0.0001
		e = 6	8.89	7.13	1.25	< 0.0001	12.6	9.93	1.27	< 0.0001
	6+	e = 6	0.160	0.0931	1.72	0.00124	0.135	0.0798	1.69	n/s
	14+	perfect	0.0035	0.000725	4.83	0.00179	0	0	n/a	n/a
		e = 10	0.0035	0.000725	4.83	0.00179	0	0	n/a	n/a
		e = 6	0.0035	0.000725	4.83	0.00179	0	0	n/a	n/a
**2**	3 to 5	perfect	4.67	3.96	1.18	< 0.0001	1.82	1.74	1.05	n/s
		e = 10	4.34	3.68	1.18	0.000266	1.78	1.71	1.04	n/s
		e = 6	6.17	5.52	1.12	0.000234	3.18	3.14	1.01	n/s
	6+	perfect	0.368	0.196	1.88	0.000248	0.0405	0.00572	7.09	n/s
		e = 10	0.599	0.356	1.68	< 0.0001	0.0720	0.0229	3.14	n/s
		e = 6	0.797	0.529	1.51	< 0.0001	0.109	0.0398	2.73	n/s
	10+	perfect	0.158	0.0606	2.62	n/s	0.0163	0.00173	9.44	< 0.0001
		e = 10	0.221	0.0931	2.38	0.00164	0.0207	0.00275	7.53	< 0.0001
		e = 6	0.252	0.109	2.32	0.00132	0.0159	0.00584	2.72	< 0.0001
**6**	6+	e = 6	0.00552	0.00341	1.62	n/s	0.0135	0.000877	15.40	< 0.0001

Of the types of microsatellite we investigated, mononucleotide runs are by far the most common, and long arrays are highly over-represented in hotspots. Although poly-A (n ≥ 6) is less than 28% enriched in hot IGRs, and is more common in non-hot than hot ORFs, poly-A (n ≥ 14) is between two and two and a half fold more common in hot IGRs, and poly-G (n ≥ 14) is nearly five fold over-represented, though this figure may be misleading as numbers of poly-G arrays are very low (Table 1). We used a lower limit of 14 bp to define long mononucleotide arrays, since a 14 bp poly-A tract was previously found to influence the activity of the *S. cerevisiae ARG4 *meiotic recombination hotspot [11]. Short poly-G runs are somewhat enriched in hotspots, and short poly-A is under-represented, but these differences can partly be explained by elevated GC content in hotspots, which has been shown previously [42], since correlations between DSB intensity and short mononucleotide runs are up to 50% weaker for IGRs, and are almost completely absent for ORFs, when controlling for GC content using partial correlation analysis (Additional file 1, Tables S3 and S4). For long microsatellites other than poly-G, correlations with DSB intensity are generally increased when controlling for GC-content (Additional file 1, Tables S3 and S4).

Dinucleotide repeats of six copies or more, and especially those with ten copies or more, are strongly associated with both hot IGRs and hot ORFs, with poly-AT the most abundant type of repeat involved (Figure 1, Table 2). Trinucleotide repeats of more than six copies are approximately twice as frequent in hot than non hot IGRs (p = 0.0027 Mann-Whitney U Test). This association is not quite significant when using the conservative Bonferroni correction for multiple hypotheses (alpha = 0.0026, see Methods section), but trinucleotide microsatellites are much scarcer than mono- or dinucleotide repeats in the yeast genome (Table 1), so statistical power to detect effects on their distribution is lower.

More marginal associations are present for some other repeat types. Long hexanucleotide microsatellites are many fold more frequent in hot than non-hot ORFs (p < 0.0001, Mann-Whitney U Test; Table 2), but this should be considered in view of the very small numbers of hexanucleotide repeats throughout the genome (Table 1). Dinucleotide repeats with between three and five copies are also significantly over-represented in hot compared with non hot IGRs, but levels of enrichment are much lower than for longer microsatellites (Table 2). Frequency of two-copy repeats is not significantly different in hot compared with non hot regions, despite the great abundance of these repeats relative to longer microsatellites, and the consequent high statistical power. Tetra- and pentanucleotide microsatellites show no significant associations at all, but these repeat types are relatively very rare throughout the yeast genome (Table 1).

### Properties of hotspot-associated microsatellites

We examined repeat array length and purity (number of mismatches with respect to the consensus repeated motif) for microsatellites of at least six copies in hotspots and other regions of the yeast genome. In addition, we compared the frequencies of insertion, substitution and deletion mismatches, with respect to the consensus repeated motifs, between hotspot-associated microsatellites and those in other regions. We found that poly-A and poly-G arrays are significantly longer in hot IGRs, and mismatched dinucleotide repeats of at least six copies are significantly longer in hot ORFs, but we saw no other significant differences in repeat length (Additional file 1, Tables S7 and S8). Microsatellites in hot and non-hot regions do not differ significantly in purity, but dinucleotide repeats in non-hot regions do show an elevated proportion of deletion mismatches (p = 0.0006, Mann-Whitney U test).

We looked the sequence motifs of all microsatellites with repeat period between three and six to see if any particular motifs were associated with hotspots. No obvious associations were seen, but we did note that poly-purine/poly-pyrimidine motifs with only one G or C are clearly over-represented among the most common motifs for low copy repeats in both hot and non-hot regions (Additional file 1, Tables S9–S12). This is likely to be related to the enrichment of poly-purine/poly-pyrimidine tracts (PPTs) in the genome as a whole [56], and, as we have reported previously, PPTs with internal tandem repeats comprise only a small proportion of total PPTs [57]. The GC-content of all repeats with at least six copies is strikingly low in IGRs throughout the genome, but there are no significant differences between hot and non-hot regions for microsatellite GC-content (Additional file 1, Tables S5 and S6).

### Possible complicating factors

The influence of microsatellites on transcriptional frequency [4-10], and the mutagenic effect of transcription on microsatellites [58] suggested that factors relating to gene expression could affect microsatellite distribution. Theoretically, this could drive the association between microsatellites and recombination hotspots in yeast, since transcriptional frequency (vegetative cells [59]) correlates with DSB intensity (p < 0.0001). However, looking at the "hottest" regions for transcriptional frequency (in equivalent numbers to the numbers of recombination hot regions studied), we found that the number of these that overlap with recombination hotspots is lower than random expectation, and the correlations between DSB intensity and frequency of microsatellites change very little when controlling for transcriptional frequency in partial correlation analysis (Additional file 1, Tables S3 and S4). DSBs have been shown to be more frequent in IGRs with two promoters (divergent transcription of flanking genes) than those with one (parallel transcription of flanking genes) or none (convergent transcription of flanking genes) [42]. We found that densities of some types of microsatellite do differ between IGRs with different numbers of promoters (Table S13). Significant differences are not present for longer microsatellites, however, with the exception of dinucleotide repeats, which are more common in IGRs with no promoters, though not significantly so when testing hot IGRs only. The association between poly-A and hotspots is not due to factors relating to the poly-A adenylation signal present in 3' untranslated regions (UTRs), since the level of enrichment of poly-A in hot over non hot IGRs does not differ by more than 5% between regions with zero, one and two promoters (two, one and zero 3' UTRs respectively).

Another factor that could complicate the association between hotspots and microsatellites is complex (tightly bunched or highly degenerate) repeats. Our initial analysis left open this possibility, since our repeat-finding algorithm does not allow multiple consecutive mismatches within single microsatellites. We therefore looked at numbers of repeats within five and ten bp of other repeats, and compared levels between hot and non-hot regions (Additional file 1, Tables S14 and S15). We found that numbers of microsatellites within complex repeats in IGRs are similar in hot and non-hot, or somewhat higher in non-hot, regions. Degenerate or complex repeats do not, therefore, affect the association between microsatellites and hot IGRs. In ORFs, complex repeats are generally somewhat more frequent in hot regions, however, and this is the case for one repeat type that showed significant over-abundance in hot ORFs, namely long dinucleotide repeats of at least six and at least ten copies. This raised the question of whether the association between this type of repeat and hot ORFs is due to the presence of highly mismatched repeats counted multiple times by our repeat finder. We therefore repeated the analysis with dinuclceotide microsatellites in ORFs occurring within 5 bp of other dinucleotide microsatellites grouped together as single arrays. This did not change the results for repeats with at least 10 copies, which still showed a strong association with hot ORFs (p < 0.0001). It did, however, reduce the significance of the association between dinucleotide arrays of at least six copies and hotspots, raising the p value to 0.014, which is above our alpha level. In view of this result, we removed dinucleotide repeats with at least six copies from our list of repeat types associated with hot ORFs.

### Microsatellite frequencies in hotspot flanking regions

We reported previously that PPTs are enriched in hotspot flanking regions as far as two ORFs removed from hotspots [57]. We repeated the analysis for microsatellites, but found no consistent evidence for a similar regional enrichment (Additional file 1, Tables S16 and S17). This suggests that the association with recombination hotspots is less broad in scale for microsatellites than for PPTs. It is also possible, however, that the lower relative abundance of microsatellites could obscure a more general broad scale association than we were able to detect, since several repeat types have higher mean frequencies in hotspot flanking regions but are too sparse for statistical significance. Furthermore, since microsatellites are enriched in both hot IGRs and hot ORFs as defined by the DSB map by Gerton *et al*., [42], and recombination breakpoints mapped on the finest possible scale are concentrated almost entirely in IGRs in yeast [55], the relationship between microsatellites and recombination probably is distal to some degree.

## Discussion

The level of enrichment of microsatellites in yeast recombination hotspots we have detailed here is considerably greater than has been seen for human hotspots [43,45]. It is not clear why this should be the case, but it is notable that the association between microsatellites and recombination in mammals is quite marked when considering broad scales of several hundred thousand kilo bases or more [49,50]. In view of evidence that humans and chimpanzees do not share a large proportion of hotspot locations in common [60,61], one explanation for the discrepancy could be that hotspots do not stay in one place long enough, in these species, to leave strong local imprints in the form of simple sequences generated by hotspot-associated factors, but that hotspot density is more constant on a larger scale. Lower lability of yeast hotspots in evolutionary time could therefore, in theory, have resulted in the stronger associations we have seen.

A better-characterized difference between the yeast and human genomes, which could also contribute to the difference between the two species in the level of association between hotspots and microsatellite abundance, is the vastly greater amount of non-coding DNA in humans. Yeast intergenic regions are small, averaging only just over 500 bp, and 75% of them contain promoters. Potentially, this could complicate the association between recombination hotspots and microsatellites due to the links between microsatellites, transcription, and recombination. Our findings suggest that this is not the case, however. It is also unlikely that transposable, or known repetitive, elements mediate the link between recombination hotspots and microsatellites in yeast, since they are not over-represented in the yeast hotspots we studied [42].

The two most obvious factors that could contribute to the association are a mutation bias, relating to recombination, or some other property of hotspot regions, causing microsatellites to form and grow, and regulation of hotspot locations by simple sequences. We attempted to isolate evidence for a mutagenic effect of recombination on microsatellites by investigating short arrays, as these are not likely to be significantly effected by replication slippage, and there is no available evidence to suggest that they have the potential to stimulate recombination. We did not find strong associations with hotspots for low-copy repeats, however, and previous evidence suggests that long microsatellites have the potential to stimulate recombination, as well as to be mutated by it. Some previous findings have cast doubt on the possibility that these phenomena have a widespread influence, and this has limited the amount of attention they have so far been given, but other evidence, including our results, suggests that they should be tested further.

Evidence that microsatellites could play a role in regulating recombination has been found at a chromosomal level in *S*.*cerevisiae *for poly-A [11], poly-AC [12,14] and pentanucleotide [13] arrays, and using extra-chromosomal DNA molecules for several repeat types [62-66]. The existence of hotspots without local microsatellites does not rule out a functional role for the sequences in recombination, since it has been established that mechanisms of hotspot regulation are heterogeneous [46,48,67]. High frequencies of microsatellites in some regions outside hotspots are also not conclusive evidence against their functional involvement, since the control of hotspot location has been shown to be complex and multi-levelled, with local and distal sequences, transcription factor binding and chromatin structure alterations all implicated (reviewed in [46,48,67]). The ability of microsatellites to bind transcription factors [68], and to affect chromatin structure *in vitro *[69] and *in vivo *[70], therefore suggest two ways in which they could function to potentiate recombination at a subset of hotspots. This could happen without DSBs actually occurring in microsatellites; deletion of a 14 bp poly-A tract reduced activity of the yeast *ARG4 *hotspot by 75% despite the fact that DSBs avoid poly-A [71,72].

It is also plausible that recombination is involved in some proportion of microsatellite mutations. The vast, presently unexplained, differences in mutation rates between loci (reviewed in [30,73]) suggest the involvement of heterogeneous mutational mechanisms or regional mutation biases. In model organisms, evidence has been found both for [12,33,74] and against [34,35] a role for recombination, in the mutation of different types of microsatellite. Studies have shown microsatellite mutation rates on the human Y Chromosome to be similar to autosomal levels [36-38], but concluding from these that recombination does not play a role in microsatellite evolution is problematic, since the Y chromosome undergoes intramolecular recombination [75]. It is therefore possible that meiotic recombination, or other properties of its hotspots, could contribute to the variability in microsatellite mutation rates at different chromosomal locations. Although unequal crossing over, or meiotic gene conversion (recombination without exchange of flanking markers), are the most obvious mechanisms for this, other factors could be important, such as replication pausing, which has been linked to microsatellite mutations [76,77], and may be causally involved in a subset of recombination hotspots [67].

## Conclusion

We found that high-copy, short-motif microsatellites are strongly associated with *S. cerevisiae *meiotic recombination hotspots. The association is weak or absent for low-copy repeats. Our results add to the weight of evidence in favour of further studying the link between microsatellites and recombination hotspots. Large-scale experimental studies in yeast could be used to quantify the level of influence hotspots have on microsatellite evolution, and to explore the possible functional role of microsatellites in regulating recombination. This work could include tracking microsatellite mutations in mono-clonal yeast populations from recombining and non recombining strains. The effect on recombination frequency of deleting microsatellites from hotspots could also be tested.

## Methods

Figures for transcriptional activity were from the study by Holstege and co-workers (1998) who mapped transcription frequency in vegetative cells for each yeast ORF [59]. For IGRs, we took the mean of the two adjacent ORFs.

### Detection of microsatellites

We detected microsatellites in the yeast genome using an algorithm written in C [78]. The programme initially generated databases of all non-overlapping repeats of two copies or greater for repeated motif sizes two to six bp, and three copies or greater for mononucleotide arrays. Separate databases were created for perfect repeats, arrays with a maximum of one mismatch allowed per ten bp of repeat sequence, and arrays with a maximum of one mismatch per six bp. Microsatellites overlapping two regions were excluded from the analysis. This occurred for less than one percent of arrays overall.

### Categorization of microsatellites

Copy number groups were two, three to five, six or more and ten or more. For mononucleotide repeats, we used 14 or more instead of ten or more, since a 14 bp poly-A tract has been shown to be a functional component of a yeast recombination hotspot [11]. We divided mononucleotide microsatellites into the equivalent motif groups A/T and G/C. Dinculeotide microsatellites were considered as a whole for statistical comparisons, but we divided them into the motif groups AT/TA, AC/CA/TG/GT, AG/GA/TC/CT and CG/GC in order to see the relative abundance of each motif type within the class. We examined sequence motifs of microsatellites with three to six bp motifs visually. We investigated compound and highly degenerate microsatellites by looking at numbers of arrays within five or ten bp of another microsatellite of the same or larger copy number group.

### Statistical analysis

Statistical comparison of means (Student's T-test and Mann-Whitney U Test, 2-tailed tests in call cases) and correlation analyses (Spearman's Rho) were done using SPSS or SAS. We initially tested the distribution of each sample for normality (Kolmogorov-Smirnov Test) and subjected significantly non-normal samples only to non-parametric tests. Because repeats were divided into 19 physically independent categories for statistical testing, Bonferroni's correction was used to set the alpha level at 0.05/19 = 0.0026. For the purpose of this calculation, the number of categories did not include different mismatch types, because, within motif and size classes, these overlap substantially so are not independent from each other. For the same reason, the size class six copies and longer was not considered to be independent of the class 10 copies and longer for the purpose of calculating the number of independent categories. Bonferroni's correction is clearly very conservative for this study, because we lose statistical power with increasing numbers of categories due to the fact that there are proportionally fewer microsatellites in each category. We would therefore gain a large amount of power by limiting the categorization to a 4-way division of microsatellites into short and long mononucleotide repeats, and short and long 2–6 bp motif repeats. This would not change the main conclusions of the paper, because, for all motif lengths, long microsatellites are either more frequent in hotspots or are extremely rare (Additional file 1, Tables S1 and S2). Some interesting information would be lost with this scheme, since poly-A and dinucleotide repeats are highly predominant among long microsatellites, and two-copy repeats are vastly more frequent than 3–5 copy repeats (Additional file 1, Tables S1 and S2), so we favoured the 19-way division.

## Authors' contributions

ATMB conceived and designed the experiments, analyzed the data and wrote the paper. JPWP wrote the computer programme. NJG contributed to the interpretation of the data and the writing of the paper. All authors read and approved the final manuscript.

## Supplementary Material

Additional file 1 bagshaw et al version 5 supplement. Supplemental tables S1–S17. Click here for file
