# Supplementary material for: High frequency of microsatellites in S. cerevisiae meiotic recombination hotspots
Source: BMC Genomics. 2008 Jan 28;9:49. doi: 10.1186/1471-2164-9-49 (PMC2267716; doi:10.1186/1471-2164-9-49)
Supplement: Additional file 1 — bagshaw et al version 5 supplement. Supplemental tables S1–S17. [file 1471-2164-9-49-S1.doc]

**Table S1:** Mean microsatellite repeat frequencies in intergenic regions (IGRs) throughout the *S. cerevisiae* genome. IGRs were divided by recombination (double-strand break) intensity as reported by Gerton and co-workers [1] into 473 hot, 89 cold and 5431 other regions, which were all IGRs not categorized as either hot or cold. The e value denotes the number of bases in any part of a repeat within which no more than one mismatch was allowed with respect to the consensus repeated motif. All p values <0.01 are shown, but caution is recommended in view of the multiple hypotheses being tested.

| **Repeat type** | | | **Mean frequency by** IGR type | | | **P value** | |
| --- | --- | --- | --- | --- | --- | --- | --- |
| Motif  length | Copy  number | Mismatch  type |  |  |  | Hot v  non-hot | Cold v  other |
|  |  |  | Hot | Other | Cold |  |  |
| 1 (A) | 3 to 5 | perfect | 35.0 | 39.9 | 39.5 | < 0.0001 | n/s |
|  |  | e=10 | 34.3 | 39.4 | 39.0 | < 0.0001 | n/s |
|  |  | e=6 | 31.8 | 36.7 | 36.6 | < 0.0001 | n/s |
|  | 6+ | perfect | 5.42 | 4.63 | 3.90 | < 0.0001 | n/s |
|  |  | e=10 | 5.24 | 4.51 | 3.87 | < 0.0001 | n/s |
|  |  | e=6 | 6.12 | 5.54 | 4.86 | 0.00173 | n/s |
|  | 14+ | perfect | 0.418 | 0.172 | 0.0663 | < 0.0001 | n/s |
|  |  | e=10 | 0.733 | 0.315 | 0.0841 | < 0.0001 | n/s |
|  |  | e=6 | 0.854 | 0.382 | 0.0841 | < 0.0001 | n/s |
| 1 (G) | 3 to 5 | perfect | 9.18 | 7.26 | 6.47 | < 0.0001 | n/s |
|  |  | e=10 | 9.16 | 7.25 | 6.49 | < 0.0001 | n/s |
|  |  | e=6 | 8.89 | 7.14 | 6.48 | < 0.0001 | n/s |
|  | 6+ | perfect | 0.118 | 0.0744 | 0.0934 | n/s | n/s |
|  |  | e=10 | 0.114 | 0.0731 | 0.0934 | n/s | n/s |
|  |  | e=6 | 0.160 | 0.0931 | 0.0934 | 0.00124 | n/s |
|  | 14+ | perfect | 0.00350 | 0.000737 | 0 | 0.00179 | n/s |
|  |  | e=10 | 0.00350 | 0.000737 | 0 | 0.00179 | n/s |
|  |  | e=6 | 0.00350 | 0.000737 | 0 | 0.00179 | n/s |
| 2 (AT) | 2 | perfect | 7.687 | 9.243 | 9.82 | < 0.0001 | n/s |
|  |  | e=10 | 7.568 | 9.102 | 9.68 | < 0.0001 | n/s |
|  |  | e=6 | 6.261 | 7.593 | 8.39 | < 0.0001 | n/s |
|  | 3 to 5 | perfect | 2.685 | 2.644 | 2.09 | n/s | n/s |
|  |  | e=10 | 2.420 | 2.481 | 2.03 | n/s | n/s |
|  |  | e=6 | 3.028 | 3.272 | 3.22 | n/s | n/s |
|  | 6+ | perfect | 0.308 | 0.178 | 0.0398 | 0.00172 | n/s |
|  |  | e=10 | 0.450 | 0.242 | 0.0989 | < 0.0001 | n/s |
|  |  | e=6 | 0.627 | 0.460 | 0.174 | 0.00155 | n/s |
|  | 10+ | perfect | 0.142 | 0.0575 | 0 | n/s | n/s |
|  |  | e=10 | 0.197 | 0.0898 | 0.00404 | 0.00586 | n/s |
|  |  | e=6 | 0.221 | 0.102 | 0.00404 | 0.00202 | n/s |
| 2 (AC) | 2 | perfect | 6.80 | 6.60 | 6.48 | n/s | n/s |
|  |  | e=10 | 6.69 | 6.55 | 6.40 | n/s | n/s |
|  |  | e=6 | 6.13 | 6.01 | 5.95 | n/s | n/s |
|  | 3 to 5 | perfect | 0.908 | 0.584 | 0.772 | < 0.0001 | n/s |
|  |  | e=10 | 0.924 | 0.569 | 0.772 | < 0.0001 | n/s |
|  |  | e=6 | 1.32 | 0.979 | 1.04 | 0.000335 | n/s |
|  | 6+ | perfect | 0.0518 | 0.0155 | 0 | n/s | n/s |
|  |  | e=10 | 0.0772 | 0.0211 | 0 | 0.00653 | n/s |
|  |  | e=6 | 0.134 | 0.0453 | 0.0499 | 0.000973 | n/s |

# Table S1: continued

| **Repeat type** | | | **Mean frequency by** IGR type | | | **P value** | |
| --- | --- | --- | --- | --- | --- | --- | --- |
| Motif  length | Copy  number | Mismatch  type |  |  |  | Hot v  non-hot | Cold v  other |
|  |  |  | Hot | Other | Cold |  |  |
| 2 (AC) | 10+ | perfect | 0.0159 | 0.00339 | 0 | n/s | n/s |
|  |  | e=10 | 0.0218 | 0.00375 | 0 | n/s | n/s |
|  |  | e=6 | 0.0283 | 0.00758 | 0 | n/s | n/s |
| 2 (AG) | 2 | perfect | 7.57 | 7.03 | 7.21 | n/s | n/s |
|  |  | e=10 | 7.53 | 7.01 | 7.19 | n/s | n/s |
|  |  | e=6 | 6.73 | 6.32 | 6.76 | n/s | n/s |
|  | 3 to 5 | perfect | 0.940 | 0.653 | 0.635 | 0.000249 | n/s |
|  |  | e=10 | 0.918 | 0.641 | 0.635 | 0.000261 | n/s |
|  |  | e=6 | 1.61 | 1.15 | 0.826 | < 0.0001 | n/s |
|  | 6+ | perfect | 0.00828 | 0.00381 | 0.0207 | n/s | n/s |
|  |  | e=10 | 0.0196 | 0.00960 | 0.0207 | n/s | n/s |
|  |  | e=6 | 0.0354 | 0.0267 | 0.0914 | n/s | 0.00391 |
|  | 10+ | perfect | 0 | 0.00065 | 0 | n/s | n/s |
|  |  | e=10 | 0.00307 | 0.00108 | 0 | n/s | n/s |
|  |  | e=6 | 0.00307 | 0.00120 | 0 | n/s | n/s |
| 2 (CG) | 2 | perfect | 1.76 | 1.43 | 1.12 | 0.000294 | n/s |
|  |  | e=10 | 1.76 | 1.43 | 1.12 | 0.000263 | n/s |
|  |  | e=6 | 1.64 | 1.38 | 1.06 | 0.00149 | n/s |
|  | 3 to 5 | perfect | 0.132 | 0.0810 | 0.112 | n/s | n/s |
|  |  | e=10 | 0.132 | 0.0809 | 0.112 | n/s | n/s |
|  |  | e=6 | 0.213 | 0.122 | 0.112 | < 0.0001 | n/s |
|  | 6+ | perfect | 0 | 0 | 0 | n/s | n/s |
|  |  | e=10 | 0 | < 0.0001 | 0 | n/s | n/s |
|  |  | e=6 | 0 | < 0.0001 | 0 | n/s | n/s |
|  | 10+ | perfect | 0 | 0 | 0 | n/s | n/s |
|  |  | e=10 | 0 | 0 | 0 | n/s | n/s |
|  |  | e=6 | 0 | 0 | 0 | n/s | n/s |
| 2 (all  motifs) | 2 | perfect | 23.8 | 24.3 | 24.6 | n/s | n/s |
|  |  | e=10 | 23.5 | 24.1 | 24.4 | n/s | n/s |
|  |  | e=6 | 20.8 | 21.3 | 22.2 | n/s | n/s |
|  | 3 to 5 | perfect | 4.67 | 3.96 | 3.61 | < 0.0001 | n/s |
|  |  | e=10 | 4.34 | 3.69 | 3.55 | 0.000266 | n/s |
|  |  | e=6 | 6.17 | 5.52 | 5.20 | 0.000234 | n/s |
|  | 6+ | perfect | 0.368 | 0.198 | 0.0605 | 0.000248 | n/s |
|  |  | e=10 | 0.599 | 0.360 | 0.125 | < 0.0001 | n/s |
|  |  | e=6 | 0.797 | 0.532 | 0.316 | < 0.0001 | n/s |
|  | 10+ | perfect | 0.158 | 0.0615 | 0 | n/s | n/s |
|  |  | e=10 | 0.221 | 0.0946 | 0.00404 | 0.00164 | n/s |
|  |  | e=6 | 0.252 | 0.110 | 0.00404 | 0.00132 | n/s |
| 3 (all  motifs) | 2 | perfect | 10.9 | 11.3 | 11.3 | n/s | n/s |
|  |  | e=10 | 10.8 | 11.1 | 11.2 | n/s | n/s |
|  |  | e=6 | 9.29 | 9.72 | 9.98 | n/s | n/s |
|  | 3 to 5 | perfect | 0.664 | 0.552 | 0.403 | n/s | n/s |
|  |  | e=10 | 0.609 | 0.528 | 0.396 | n/s | n/s |
|  |  | e=6 | 1.97 | 1.93 | 1.32 | n/s | n/s |

**Table S1:** continued

| **Repeat type** | | | **Mean frequency by** IGR type | | | **P value** | |
| --- | --- | --- | --- | --- | --- | --- | --- |
| Motif  length | Copy  number | Mismatch  type |  |  |  | Hot v  non-hot | Cold v  other |
|  |  |  | Hot | Other | Cold |  |  |
| 3 (all  motifs) | 6+ | perfect | 0.0460 | 0.0126 | 0 | n/s | n/s |
|  |  | e=10 | 0.0627 | 0.0291 | 0 | n/s | n/s |
|  |  | e=6 | 0.109 | 0.0533 | 0 | 0.00270 | n/s |
|  | 10+ | perfect | 0.00970 | 0.00559 | 0 | n/s | n/s |
|  |  | e=10 | 0.0215 | 0.0105 | 0 | n/s | n/s |
|  |  | e=6 | 0.0215 | 0.0116 | 0 | n/s | n/s |
| 4 (all  motifs) | 2 | perfect | 4.36 | 3.97 | 4.17 | n/s | n/s |
|  |  | e=10 | 4.17 | 3.77 | 3.95 | n/s | n/s |
|  |  | e=6 | 3.44 | 3.04 | 3.28 | n/s | n/s |
|  | 3 to 5 | perfect | 0.151 | 0.107 | 0.118 | 0.00311 | n/s |
|  |  | e=10 | 0.274 | 0.286 | 0.248 | n/s | n/s |
|  |  | e=6 | 0.329 | 0.368 | 0.402 | n/s | n/s |
|  | 6+ | perfect | 0 | 0.00509 | 0 | n/s | n/s |
|  |  | e=10 | 0 | 0.00589 | 0 | n/s | n/s |
|  |  | e=6 | 0 | 0.00865 | 0 | n/s | n/s |
|  | 10+ | perfect | 0 | 0.00157 | 0 | n/s | n/s |
|  |  | e=10 | 0 | 0.00157 | 0 | n/s | n/s |
|  |  | e=6 | 0 | 0.00157 | 0 | n/s | n/s |
| 5 (all  motifs) | 2 | perfect | 1.72 | 1.58 | 1.38 | n/s | n/s |
|  |  | e=10 | 1.63 | 1.45 | 1.34 | 0.102 | n/s |
|  |  | e=6 | 1.28 | 1.12 | 1.04 | n/s | n/s |
|  | 3 to 5 | perfect | 0.0482 | 0.0356 | 0.0205 | n/s | n/s |
|  |  | e=10 | 0.0999 | 0.103 | 0.0732 | n/s | n/s |
|  |  | e=6 | 0.0867 | 0.0992 | 0.0732 | n/s | n/s |
|  | 6+ | perfect | 0 | 0.00082 | 0 | n/s | n/s |
|  |  | e=10 | 0.00137 | 0.00106 | 0 | n/s | n/s |
|  |  | e=6 | 0.00137 | 0.00267 | 0 | n/s | n/s |
|  | 10+ | perfect | 0 | 0 | 0 | n/s | n/s |
|  |  | e=10 | 0 | 0 | 0 | n/s | n/s |
|  |  | e=6 | 0 | 0 | 0 | n/s | n/s |
| 6 (all  motifs) | 2 | perfect | 0.811 | 0.654 | 0.564 | n/s | n/s |
|  |  | e=10 | 0.727 | 0.575 | 0.534 | n/s | n/s |
|  |  | e=6 | 0.518 | 0.394 | 0.363 | n/s | n/s |
|  | 3 to 5 | perfect | 0.0491 | 0.0233 | 0.0109 | n/s | n/s |
|  |  | e=10 | 0.0509 | 0.0440 | 0.0412 | n/s | n/s |
|  |  | e=6 | 0.0409 | 0.0290 | 0.0412 | n/s | n/s |
|  | 6+ | perfect | 0.00552 | 0.000628 | 0 | n/s | n/s |
|  |  | e=10 | 0.00552 | 0.00459 | 0 | n/s | n/s |
|  |  | e=6 | 0.00552 | 0.00347 | 0 | n/s | n/s |
|  | 10+ | perfect | 0 | 0 | 0 | n/s | n/s |
|  |  | e=10 | 0 | 0.00181 | 0 | n/s | n/s |
|  |  | e=6 | 0 | 0.00089 | 0 | n/s | n/s |

**Table S2:** Mean microsatellite repeat frequencies in open reading frames (ORFs) throughout the *S. cerevisiae* genome. ORFs were divided by recombination (double-strand break) intensity as reported by Gerton and co-workers [1] into 297 hot, 49 cold and 5634 other regions, which were all ORFs not categorized as either hot or cold. The e value denotes the number of bases in any part of a repeat within which no more than one mismatch was allowed with respect to the consensus repeated motif. All p values <0.01 are shown, but caution is recommended in view of the multiple hypotheses being tested.

| **Repeat type** | | | **Mean frequency by** ORF type | | | **P value** | |
| --- | --- | --- | --- | --- | --- | --- | --- |
| Motif  length | Copy  number | Mismatch  type |  |  |  | Hot v  non-hot | Cold v  other |
|  |  |  | Hot | Other | Cold |  |  |
| 1 (A) | 3 to 5 | perfect | 29.2 | 36.1 | 35.8 | < 0.0001 | n/s |
|  |  | e=10 | 29.1 | 36.0 | 35.8 | < 0.0001 | n/s |
|  |  | e=6 | 28.0 | 34.7 | 34.5 | < 0.0001 | n/s |
|  | 6+ | perfect | 0.981 | 1.64 | 1.51 | < 0.0001 | n/s |
|  |  | e=10 | 0.978 | 1.64 | 1.51 | < 0.0001 | n/s |
|  |  | e=6 | 1.28 | 2.13 | 1.92 | < 0.0001 | n/s |
|  | 14+ | perfect | 0.0134 | 0.00740 | 0 | n/s | n/s |
|  |  | e=10 | 0.0182 | 0.0167 | 0 | n/s | n/s |
|  |  | e=6 | 0.0218 | 0.0273 | 0.00477 | n/s | n/s |
| 1 (G) | 3 to 5 | perfect | 12.9 | 10.1 | 10.7 | < 0.0001 | n/s |
|  |  | e=10 | 12.9 | 10.1 | 10.7 | < 0.0001 | n/s |
|  |  | e=6 | 12.6 | 9.92 | 10.6 | < 0.0001 | n/s |
|  | 6+ | perfect | 0.0992 | 0.0631 | 0.0586 | n/s | n/s |
|  |  | e=10 | 0.0992 | 0.0631 | 0.0586 | n/s | n/s |
|  |  | e=6 | 0.135 | 0.0796 | 0.0996 | n/s | n/s |
|  | 14+ | perfect | 0 | 0 | 0 | n/a | n/s |
|  |  | e=10 | 0 | 0 | 0 | n/a | n/s |
|  |  | e=6 | 0 | 0 | 0 | n/a | n/s |
| 2 (AT) | 2 | perfect | 4.46 | 5.91 | 6.36 | < 0.0001 | n/s |
|  |  | e=10 | 4.43 | 5.89 | 6.35 | < 0.0001 | n/s |
|  |  | e=6 | 4.07 | 5.37 | 5.68 | < 0.0001 | n/s |
|  | 3 to 5 | perfect | 0.434 | 0.581 | 0.539 | 0.000118 | n/s |
|  |  | e=10 | 0.409 | 0.576 | 0.522 | < 0.0001 | n/s |
|  |  | e=6 | 0.655 | 0.966 | 1.04 | < 0.0001 | n/s |
|  | 6+ | perfect | 0.0244 | 0.00364 | 0 | < 0.0001 | n/s |
|  |  | e=10 | 0.0356 | 0.00649 | 0 | < 0.0001 | n/s |
|  |  | e=6 | 0.0442 | 0.0171 | 0 | n/s | n/s |
|  | 10+ | perfect | 0.00970 | 0.00109 | 0 | 0.000311 | n/s |
|  |  | e=10 | 0.0141 | 0.00167 | 0 | < 0.0001 | n/s |
|  |  | e=6 | 0.00926 | 0.00209 | 0 | < 0.0001 | n/s |
| 2 (AC) | 2 | perfect | 8.21 | 7.05 | 7.66 | < 0.0001 | n/s |
|  |  | e=10 | 8.18 | 7.03 | 7.65 | < 0.0001 | n/s |
|  |  | e=6 | 7.50 | 6.50 | 7.00 | < 0.0001 | n/s |
|  | 3 to 5 | perfect | 0.589 | 0.419 | 0.309 | 0.00522 | n/s |
|  |  | e=10 | 0.573 | 0.417 | 0.309 | 0.00788 | n/s |
|  |  | e=6 | 1.10 | 0.814 | 0.896 | < 0.0001 | n/s |
|  | 6+ | perfect | 0.00661 | 0.00162 | 0 | n/s | n/s |
|  |  | e=10 | 0.022185 | 0.00204 | 0 | 0.00137 | n/s |
|  |  | e=6 | 0.035799 | 0.00995 | 0 | n/s | n/s |

**Table S2:** continued

| **Repeat type** | | | **Mean frequency by** ORF type | | | **P value** | |
| --- | --- | --- | --- | --- | --- | --- | --- |
| Motif  length | Copy  number | Mismatch  type |  |  |  | Hot v  non-hot | Cold v  other |
|  |  |  | Hot | Other | Cold |  |  |
| 2 (AC) | 10+ | perfect | 0.00661 | 0.000137 | 0 | n/s | n/s |
|  |  | e=10 | 0.00661 | 0.000522 | 0 | n/s | n/s |
|  |  | e=6 | 0.00661 | 0.00321 | 0 | n/s | n/s |
| 2 (AG) | 2 | perfect | 9.20 | 8.80 | 8.60 | n/s | n/s |
|  |  | e=10 | 9.15 | 8.77 | 8.58 | n/s | n/s |
|  |  | e=6 | 8.31 | 8.00 | 8.18 | n/s | n/s |
|  | 3 to 5 | perfect | 0.695 | 0.707 | 0.517 | n/s | n/s |
|  |  | e=10 | 0.694 | 0.702 | 0.517 | n/s | n/s |
|  |  | e=6 | 1.26 | 1.29 | 0.917 | n/s | n/s |
|  | 6+ | perfect | 0.00954 | 0.000512 | 0 | 0.00338 | n/s |
|  |  | e=10 | 0.00954 | 0.00174 | 0 | n/s | n/s |
|  |  | e=6 | 0.0285 | 0.0129 | 0 | n/s | n/s |
|  | 10+ | perfect | 0 | 0.000512 | 0 | n/s | n/s |
|  |  | e=10 | 0 | 0.000584 | 0 | n/s | n/s |
|  |  | e=6 | 0 | 0.000584 | 0 | n/s | n/s |
| 2 (CG) | 2 | perfect | 2.11 | 1.26 | 1.18 | < 0.0001 | n/s |
|  |  | e=10 | 2.10 | 1.26 | 1.18 | < 0.0001 | n/s |
|  |  | e=6 | 2.01 | 1.21 | 1.12 | < 0.0001 | n/s |
|  | 3 to 5 | perfect | 0.106 | 0.0342 | 0.0251 | 0.000409 | n/s |
|  |  | e=10 | 0.106 | 0.0342 | 0.0251 | 0.000409 | n/s |
|  |  | e=6 | 0.161 | 0.0671 | 0.0479 | < 0.0001 | n/s |
|  | 6+ | perfect | 0 | 0 | 0 | n/s | n/s |
|  |  | e=10 | 0 | 0 | 0 | n/s | n/s |
|  |  | e=6 | 0 | 0.000137 | 0 | n/s | n/s |
|  | 10+ | perfect | 0 | 0 | 0 | n/s | n/s |
|  |  | e=10 | 0 | 0 | 0 | n/s | n/s |
|  |  | e=6 | 0 | 0 | 0 | n/s | n/s |
| 2 (all  motifs) | 2 | perfect | 24.0 | 23.0 | 23.8 | 0.00308 | n/s |
|  |  | e=10 | 23.9 | 23.0 | 23.8 | 0.00493 | n/s |
|  |  | e=6 | 21.9 | 21.1 | 22.0 | n/s | n/s |
|  | 3 to 5 | perfect | 1.82 | 1.74 | 1.39 | n/s | n/s |
|  |  | e=10 | 1.78 | 1.72 | 1.37 | n/s | n/s |
|  |  | e=6 | 3.18 | 3.14 | 2.90 | n/s | n/s |
|  | 6+ | perfect | 0.0405 | 0.00577 | 0 | < 0.0001 | n/s |
|  |  | e=10 | 0.0720 | 0.0231 | 0 | n/s | n/s |
|  |  | e=6 | 0.109 | 0.0401 | 0 | n/s | n/s |
|  | 10+ | perfect | 0.0163 | 0.00174 | 0 | < 0.0001 | n/s |
|  |  | e=10 | 0.0207 | 0.00277 | 0 | < 0.0001 | n/s |
|  |  | e=6 | 0.0159 | 0.00589 | 0 | < 0.0001 | n/s |
| 3 (all  motifs) | 2 | perfect | 13.9 | 13.6 | 14.5 | n/s | n/s |
|  |  | e=10 | 13.8 | 13.6 | 14.4 | n/s | n/s |
|  |  | e=6 | 12.4 | 12.3 | 12.8 | n/s | n/s |
|  | 3 to 5 | perfect | 0.895 | 0.684 | 0.624 | n/s | n/s |
|  |  | e=10 | 0.844 | 0.657 | 0.618 | n/s | n/s |
|  |  | e=6 | 2.03 | 1.81 | 1.81 | n/s | n/s |

**Table S2:** continued

| **Repeat type** | | | **Mean frequency by** ORF type | | | **P value** | |
| --- | --- | --- | --- | --- | --- | --- | --- |
| Motif  length | Copy  number | Mismatch  type |  |  |  | Hot v  non-hot | Cold v  other |
|  |  |  | Hot | Other | Cold |  |  |
| 3 (all  motifs) | 6+ | perfect | 0.0241 | 0.0181 | 0 | n/s | n/s |
|  |  | e=10 | 0.0572 | 0.0355 | 0 | n/s | n/s |
|  |  | e=6 | 0.0773 | 0.0539 | 0.0410 | n/s | n/s |
|  | 10+ | perfect | 0 | 0.00257 | 0 | n/s | n/s |
|  |  | e=10 | 0 | 0.00574 | 0 | n/s | n/s |
|  |  | e=6 | 0 | 0.00986 | 0 | n/s | n/s |
| 4 (all  motifs) | 2 | perfect | 2.52 | 2.74 | 2.66 | n/s | n/s |
|  |  | e=10 | 2.42 | 2.68 | 2.63 | n/s | n/s |
|  |  | e=6 | 2.15 | 2.35 | 2.21 | n/s | n/s |
|  | 3 to 5 | perfect | 0.0196 | 0.0157 | 0.00669 | n/s | n/s |
|  |  | e=10 | 0.102 | 0.0718 | 0.0353 | n/s | n/s |
|  |  | e=6 | 0.127 | 0.108 | 0.0822 | n/s | n/s |
|  | 6+ | perfect | 0 | 0.000267 | 0 | n/s | n/s |
|  |  | e=10 | 0 | 0.000267 | 0 | n/s | n/s |
|  |  | e=6 | 0 | 0.000484 | 0 | n/s | n/s |
|  | 10+ | perfect | 0 | 0 | 0 | n/s | n/s |
|  |  | e=10 | 0 | 0 | 0 | n/s | n/s |
|  |  | e=6 | 0 | 0 | 0 | n/s | n/s |
| 5 (all  motifs) | 2 | perfect | 0.766 | 0.865 | 0.830 | n/s | n/s |
|  |  | e=10 | 0.755 | 0.836 | 0.811 | n/s | n/s |
|  |  | e=6 | 0.646 | 0.714 | 0.763 | n/s | n/s |
|  | 3 to 5 | perfect | 0 | 0.007087 | 0 | n/s | n/s |
|  |  | e=10 | 0.00467 | 0.0238 | 0.0260 | n/s | n/s |
|  |  | e=6 | 0.00219 | 0.0207 | 0.0260 | n/s | n/s |
|  | 6+ | perfect | 0 | 0 | 0 | n/a | n/s |
|  |  | e=10 | 0 | 0.000652 | 0 | n/s | n/s |
|  |  | e=6 | 0 | 0.000732 | 0 | n/s | n/s |
|  | 10+ | perfect | 0 | 0 | 0 | n/s | n/s |
|  |  | e=10 | 0 | 0 | 0 | n/s | n/s |
|  |  | e=6 | 0 | 0.000415 | 0 | n/s | n/s |
| 6 (all  motifs) | 2 | perfect | 0.618 | 0.499 | 0.341 | n/s | n/s |
|  |  | e=10 | 0.575 | 0.472 | 0.319 | n/s | n/s |
|  |  | e=6 | 0.480 | 0.408 | 0.258 | n/s | n/s |
|  | 3 to 5 | perfect | 0.0495 | 0.0247 | 0.0177 | n/s | n/s |
|  |  | e=10 | 0.0627 | 0.0282 | 0.0332 | n/s | n/s |
|  |  | e=6 | 0.0616 | 0.0201 | 0.0158 | n/s | n/s |
|  | 6+ | perfect | 0 | 0.000205 | 0 | n/s | n/s |
|  |  | e=10 | 0.00839 | 0.00286 | 0 | n/s | n/s |
|  |  | e=6 | 0.0135 | 0.000885 | 0 | < 0.0001 | n/s |
|  | 10+ | perfect | 0 | 0 | 0 | n/a | n/s |
|  |  | e=10 | 0.00661 | < 0.0001 | 0 | 0.00336 | n/s |
|  |  | e=6 | 0.00661 | 0.000345 | 0 | n/s | n/s |

**Table S3:** Spearman correlations between DSB intensity and microsatellite repeat frequency (RF) for all IGRs in the *S. cerevisiae* genome. Partial correlations are also shown, controlling for GC content (GC), and transcriptional frequency (TF), which was the mean of the two adjacent ORFs. The e value denotes the number of bases in any part of a repeat within which no more than one mismatch was allowed with respect to the consensus repeated motif. All p values <0.01 are shown, but caution is recommended in view of the multiple hypotheses being tested.

| **Repeat type** | | | **RF v DSB intensity** | | **Controlling for GC** | | **Controlling for TF** | |
| --- | --- | --- | --- | --- | --- | --- | --- | --- |
| Motif length | Repeat  count | Mismatch  type | Coeff. | P value | Coeff. | P value | Coeff. | P value |
| 1 (A) | 3 to 5 | perfect | -0.137 | <.0001 | -0.0948 | <.0001 | -0.140 | <.0001 |
|  |  | e=10 | -0.142 | <.0001 | -0.102 | <.0001 | -0.144 | <.0001 |
|  |  | e=6 | -0.152 | <.0001 | -0.120 | <.0001 | -0.152 | <.0001 |
|  | 6+ | perfect | 0.0743 | <.0001 | 0.122 | <.0001 | 0.0615 | <.0001 |
|  |  | e=10 | 0.0726 | <.0001 | 0.119 | <.0001 | 0.0601 | <.0001 |
|  |  | e=6 | 0.0662 | <.0001 | 0.119 | <.0001 | 0.0537 | <.0001 |
|  | 14+ | perfect | 0.0716 | <.0001 | 0.0804 | <.0001 | 0.0705 | <.0001 |
|  |  | e=10 | 0.111 | <.0001 | 0.123 | <.0001 | 0.107 | <.0001 |
|  |  | e=6 | 0.114 | <.0001 | 0.127 | <.0001 | 0.110 | <.0001 |
| 1 (G) | 3 to 5 | perfect | 0.132 | <.0001 | 0.0638 | <.0001 | 0.132 | <.0001 |
|  |  | e=10 | 0.131 | <.0001 | 0.0623 | <.0001 | 0.131 | <.0001 |
|  |  | e=6 | 0.125 | <.0001 | 0.0570 | <.0001 | 0.125 | <.0001 |
|  | 6+ | perfect | 0.0380 | 0.0034 | 0.0160 | n/s | 0.0471 | 0.0004 |
|  |  | e=10 | 0.0389 | 0.0027 | 0.0169 | n/s | 0.0478 | 0.0003 |
|  |  | e=6 | 0.0586 | <.0001 | 0.0325 | n/s | 0.0652 | <.0001 |
|  | 14+ | perfect | 0.0214 | n/s | 0.0160 | n/s | 0.0197 | n/s |
|  |  | e=10 | 0.0214 | n/s | 0.0160 | n/s | 0.0197 | n/s |
|  |  | e=6 | 0.0214 | n/s | 0.0160 | n/s | 0.0197 | n/s |
| 2 (AT) | 2 | perfect | -0.118 | <.0001 | -0.0778 | <.0001 | -0.125 | <.0001 |
|  |  | e=10 | -0.124 | <.0001 | -0.0861 | <.0001 | -0.130 | <.0001 |
|  |  | e=6 | -0.115 | <.0001 | -0.0883 | <.0001 | -0.117 | <.0001 |
|  | 3 to 5 | perfect | -0.00831 | n/s | 0.0212 | n/s | -0.0136 | n/s |
|  |  | e=10 | -0.0133 | n/s | 0.0112 | n/s | -0.0171 | n/s |
|  |  | e=6 | -0.0340 | 0.0086 | -0.0005 | n/s | -0.0401 | 0.0024 |
|  | 6+ | perfect | 0.0437 | 0.0007 | 0.0555 | <.0001 | 0.0439 | 0.0009 |
|  |  | e=10 | 0.0395 | 0.0023 | 0.0511 | <.0001 | 0.0370 | 0.0052 |
|  |  | e=6 | 0.0257 | n/s | 0.0442 | 0.0007 | 0.0217 | n/s |
|  | 10+ | perfect | 0.0278 | n/s | 0.0378 | 0.0035 | 0.0290 | n/s |
|  |  | e=10 | 0.0393 | 0.0024 | 0.0501 | 0.0001 | 0.0417 | 0.0016 |
|  |  | e=6 | 0.0447 | 0.0006 | 0.0547 | <.0001 | 0.0470 | 0.0004 |
| 2 (AC) | 2 | perfect | 0.0067 | n/s | -0.0227 | n/s | 0.0124 | n/s |
|  |  | e=10 | 0.00313 | n/s | -0.0261 | n/s | 0.00894 | n/s |
|  |  | e=6 | -0.00982 | n/s | -0.0369 | 0.0044 | -0.00587 | n/s |
|  | 3 to 5 | perfect | 0.0678 | <.0001 | 0.0378 | 0.0035 | 0.0671 | <.0001 |
|  |  | e=10 | 0.0624 | <.0001 | 0.0330 | n/s | 0.0615 | <.0001 |
|  |  | e=6 | 0.0739 | <.0001 | 0.0414 | 0.0014 | 0.0745 | <.0001 |
|  | 6+ | perfect | 0.0290 | n/s | 0.0225 | n/s | 0.0327 | n/s |
|  |  | e=10 | 0.0339 | 0.0089 | 0.0260 | n/s | 0.0427 | 0.0012 |
|  |  | e=6 | 0.0579 | <.0001 | 0.0443 | 0.0006 | 0.0633 | <.0001 |

# Table S3: continued

| **Repeat type** | | | **RF v DSB intensity** | | **Controlling for GC** | | **Controlling for TF** | |  |
| --- | --- | --- | --- | --- | --- | --- | --- | --- | --- |
| Motif length | Repeat  count | Mismatch  type | Coeff. | P value | Coeff. | P value | Coeff. | P value | |
| 2 (AC) | 10+ | perfect | 0.0154 | n/s | 0.0139 | n/s | 0.0196 | n/s |  |
|  |  | e=10 | 0.0171 | n/s | 0.0151 | n/s | 0.0204 | n/s |  |
|  |  | e=6 | 0.0450 | 0.0005 | 0.0356 | 0.006 | 0.0512 | 0.0001 |  |
| 2 (AG) | 2 | perfect | 0.0264 | n/s | -0.0138 | n/s | 0.0260 | n/s |  |
|  |  | e=10 | 0.0260 | n/s | -0.0140 | n/s | 0.0255 | n/s |  |
|  |  | e=6 | 0.0188 | n/s | -0.0187 | n/s | 0.0185 | n/s |  |
|  | 3 to 5 | perfect | 0.0510 | <.0001 | 0.0213 | n/s | 0.0536 | <.0001 |  |
|  |  | e=10 | 0.0497 | 0.0001 | 0.0201 | n/s | 0.0519 | <.0001 |  |
|  |  | e=6 | 0.0677 | <.0001 | 0.0335 | 0.0097 | 0.0723 | <.0001 |  |
|  | 6+ | perfect | 0.0388 | 0.0028 | 0.0332 | n/s | 0.0369 | 0.0053 |  |
|  |  | e=10 | 0.0554 | <.0001 | 0.0478 | 0.0002 | 0.0528 | <.0001 |  |
|  |  | e=6 | 0.0472 | 0.0003 | 0.0380 | 0.0033 | 0.0476 | 0.0003 |  |
|  | 10+ | perfect | 0.0239 | n/s | 0.0226 | n/s | 0.0237 | n/s |  |
|  |  | e=10 | 0.0355 | 0.0061 | 0.0317 | n/s | 0.0363 | 0.0061 |  |
|  |  | e=6 | 0.0383 | 0.0031 | 0.0335 | 0.0097 | 0.0387 | 0.0034 |  |
| 2 (CG) | 2 | perfect | 0.0615 | <.0001 | 0.00827 | n/s | 0.0613 | <.0001 |  |
|  |  | e=10 | 0.0609 | <.0001 | 0.0077 | n/s | 0.0607 | <.0001 |  |
|  |  | e=6 | 0.0533 | <.0001 | 0.00138 | n/s | 0.0549 | <.0001 |  |
|  | 3 to 5 | perfect | 0.0537 | <.0001 | 0.0282 | n/s | 0.0606 | <.0001 |  |
|  |  | e=10 | 0.0529 | <.0001 | 0.0275 | n/s | 0.0606 | <.0001 |  |
|  |  | e=6 | 0.0787 | <.0001 | 0.0466 | 0.0003 | 0.0823 | <.0001 |  |
|  | 6+ | perfect | n/a | n/a | n/a | n/a | n/a | n/a |  |
|  |  | e=10 | 0.0153 | n/s | 0.0129 | n/s | n/a | n/a |  |
|  |  | e=6 | 0.0153 | n/s | 0.0129 | n/s | n/a | n/a |  |
|  | 10+ | perfect | n/a | n/a | n/a | n/a | n/a | n/a |  |
|  |  | e=10 | n/a | n/a | n/a | n/a | n/a | n/a |  |
|  |  | e=6 | n/a | n/a | n/a | n/a | n/a | n/a |  |
| 2 (all  motifs) | 2 | perfect | -0.0375 | 0.0038 | -0.0532 | <.0001 | -0.0372 | 0.005 |  |
|  |  | e=10 | -0.0428 | 0.001 | -0.0595 | <.0001 | -0.0425 | 0.0013 |  |
|  |  | e=6 | -0.0484 | 0.0002 | -0.0723 | <.0001 | -0.0459 | 0.0005 |  |
|  | 3 to 5 | perfect | 0.0426 | 0.001 | 0.0555 | <.0001 | 0.0390 | 0.0032 |  |
|  |  | e=10 | 0.0375 | 0.0038 | 0.0459 | 0.0004 | 0.0346 | 0.0089 |  |
|  |  | e=6 | 0.0405 | 0.0018 | 0.0508 | <.0001 | 0.0393 | 0.003 |  |
|  | 6+ | perfect | 0.0559 | <.0001 | 0.0643 | <.0001 | 0.0561 | <.0001 |  |
|  |  | e=10 | 0.0565 | <.0001 | 0.0614 | <.0001 | 0.0532 | <.0001 |  |
|  |  | e=6 | 0.0522 | <.0001 | 0.0633 | <.0001 | 0.0485 | 0.0002 |  |
|  | 10+ | perfect | 0.0341 | 0.0085 | 0.0431 | 0.0009 | 0.0361 | 0.0064 |  |
|  |  | e=10 | 0.0468 | 0.0003 | 0.0562 | <.0001 | 0.0497 | 0.0002 |  |
|  |  | e=6 | 0.0634 | <.0001 | 0.0687 | <.0001 | 0.0670 | <.0001 |  |
| 3 (all  motifs) | 2 | perfect | -0.0105 | n/s | -0.0173 | n/s | -0.0123 | n/s |  |
|  |  | e=10 | -0.0176 | n/s | -0.0261 | n/s | -0.0192 | n/s |  |
|  |  | e=6 | -0.0282 | n/s | -0.0445 | 0.0006 | -0.0282 | n/s |  |
|  | 3 to 5 | perfect | 0.0484 | 0.0002 | 0.0417 | 0.0013 | 0.0464 | 0.0005 |  |
|  |  | e=10 | 0.0460 | 0.0004 | 0.0377 | 0.0037 | 0.0439 | 0.0009 |  |
|  |  | e=6 | 0.0342 | 0.0083 | 0.0353 | 0.0065 | 0.0253 | n/s |  |

# Table S3: continued

| **Repeat type** | | | **RF v DSB intensity** | | **Controlling for GC** | | **Controlling for TF** | |  |
| --- | --- | --- | --- | --- | --- | --- | --- | --- | --- |
| Motif length | Repeat  count | Mismatch  type | Coeff. | P value | Coeff. | P value | Coeff. | P value | |
| 3 (all  motifs) | 6+ | perfect | 0.0212 | n/s | 0.0269 | n/s | 0.0238 | n/s |  |
|  |  | e=10 | 0.0312 | n/s | 0.0364 | 0.005 | 0.0306 | n/s |  |
|  |  | e=6 | 0.0489 | 0.0002 | 0.0539 | <.0001 | 0.0462 | 0.0005 |  |
|  | 10+ | perfect | -0.00896 | n/s | -0.00192 | n/s | -0.00829 | n/s |  |
|  |  | e=10 | 0.0237 | n/s | 0.0336 | 0.0096 | 0.0218 | n/s |  |
|  |  | e=6 | 0.0292 | n/s | 0.0390 | 0.0026 | 0.0268 | n/s |  |
| 4 (all  motifs) | 2 | perfect | 0.0306 | n/s | 0.0376 | 0.0037 | 0.0232 | n/s |  |
|  |  | e=10 | 0.0298 | n/s | 0.0344 | 0.0079 | 0.0230 | n/s |  |
|  |  | e=6 | 0.0170 | n/s | 0.0132 | n/s | 0.00953 | n/s |  |
|  | 3 to 5 | perfect | 0.0397 | 0.0022 | 0.0400 | 0.002 | 0.0422 | 0.0014 |  |
|  |  | e=10 | 0.0439 | 0.0007 | 0.0440 | 0.0007 | 0.0417 | 0.0016 |  |
|  |  | e=6 | 0.0463 | 0.0003 | 0.0473 | 0.0003 | 0.0424 | 0.0014 |  |
|  | 6+ | perfect | -0.00019 | n/s | 0.00273 | n/s | 0.00078 | n/s |  |
|  |  | e=10 | 0.00675 | n/s | 0.0113 | n/s | -0.00017 | n/s |  |
|  |  | e=6 | 0.0231 | n/s | 0.0257 | n/s | 0.0178 | n/s |  |
|  | 10+ | perfect | 0.00595 | n/s | 0.00966 | n/s | 0.00481 | n/s |  |
|  |  | e=10 | 0.00595 | n/s | 0.00966 | n/s | 0.00481 | n/s |  |
|  |  | e=6 | 0.00595 | n/s | 0.00966 | n/s | 0.00481 | n/s |  |
| 5 (all  motifs) | 2 | perfect | 0.0357 | 0.0059 | 0.0354 | 0.0062 | 0.0242 | n/s |  |
|  |  | e=10 | 0.0310 | n/s | 0.0279 | n/s | 0.0194 | n/s |  |
|  |  | e=6 | 0.0240 | n/s | 0.0157 | n/s | 0.0148 | n/s |  |
|  | 3 to 5 | perfect | 0.0294 | n/s | 0.0215 | n/s | 0.0324 | n/s |  |
|  |  | e=10 | 0.0366 | 0.0047 | 0.0309 | n/s | 0.0333 | n/s |  |
|  |  | e=6 | 0.0331 | n/s | 0.0280 | n/s | 0.0328 | n/s |  |
|  | 6+ | perfect | -0.0273 | n/s | -0.0274 | n/s | -0.0292 | n/s |  |
|  |  | e=10 | -0.0171 | n/s | -0.0215 | n/s | -0.0146 | n/s |  |
|  |  | e=6 | 0.00077 | n/s | 0.00032 | n/s | -0.00104 | n/s |  |
|  | 10+ | perfect | n/a | n/a | n/a | n/a | n/a | n/a |  |
|  |  | e=10 | n/a | n/a | n/a | n/a | n/a | n/a |  |
|  |  | e=6 | n/a | n/a | n/a | n/a | n/a | n/a |  |
| 6 (all  motifs) | 2 | perfect | 0.0440 | 0.0007 | 0.0386 | 0.0029 | 0.0457 | 0.0005 |  |
|  |  | e=10 | 0.0348 | 0.0072 | 0.0289 | n/s | 0.0377 | 0.0044 |  |
|  |  | e=6 | 0.0282 | n/s | 0.0157 | n/s | 0.0344 | 0.0093 |  |
|  | 3 to 5 | perfect | 0.0299 | n/s | 0.0233 | n/s | 0.0337 | n/s |  |
|  |  | e=10 | 0.0278 | n/s | 0.0234 | n/s | 0.0305 | n/s |  |
|  |  | e=6 | 0.0232 | n/s | 0.0218 | n/s | 0.0309 | n/s |  |
|  | 6+ | perfect | 0.0197 | n/s | 0.0166 | n/s | 0.0206 | n/s |  |
|  |  | e=10 | 0.0248 | n/s | 0.0131 | n/s | 0.0303 | n/s |  |
|  |  | e=6 | 0.00238 | n/s | -0.00224 | n/s | -0.00122 | n/s |  |
|  | 10+ | perfect | n/a | n/a | n/a | n/a | n/a | n/a |  |
|  |  | e=10 | 0.0130 | n/s | 0.0042 | n/s | 0.0234 | n/s |  |
|  |  | e=6 | 0.00502 | n/s | -0.00078 | n/s | 0.00154 | n/s |  |

**Table S4 :** Spearman correlations between DSB intensity and microsatellite repeat frequency (RF) for all ORFs in the *S. cerevisiae* genome. Partial correlations are also shown, controlling for GC content (GC), and transcriptional frequency (TF). The e value denotes the number of bases in any part of a repeat within which no more than one mismatch was allowed with respect to the consensus repeated motif. All p values <0.01 are shown, but caution is recommended in view of the multiple hypotheses being tested.

| Repeat type | | | **RF v DSB intensity** | | **Controlling for GC** | | **Controlling for TF** | |  |
| --- | --- | --- | --- | --- | --- | --- | --- | --- | --- |
| Motif length | Repeat  count | Mismatch  type | Coeff. | P value | Coeff. | P value | Coeff. | P value | |
| 1 (A) | 3 to 5 | perfect | -0.241 | <.0001 | -0.0430 | 0.0012 | -0.224 | <.0001 |  |
|  |  | e=10 | -0.241 | <.0001 | -0.0443 | 0.0008 | -0.224 | <.0001 |  |
|  |  | e=6 | -0.237 | <.0001 | -0.0486 | 0.0003 | -0.219 | <.0001 |  |
|  | 6+ | perfect | -0.149 | <.0001 | -0.0232 | n/s | -0.126 | <.0001 |  |
|  |  | e=10 | -0.148 | <.0001 | -0.0229 | n/s | -0.125 | <.0001 |  |
|  |  | e=6 | -0.155 | <.0001 | -0.0159 | n/s | -0.140 | <.0001 |  |
|  | 14+ | perfect | 0.0437 | 0.001 | 0.0512 | 0.0001 | 0.0355 | n/s |  |
|  |  | e=10 | 0.0307 | n/s | 0.0395 | 0.0029 | 0.0214 | n/s |  |
|  |  | e=6 | 0.0095 | n/s | 0.0243 | n/s | 0.00101 | n/s |  |
| 1 (G) | 3 to 5 | perfect | 0.141 | <.0001 | 0.00175 | n/s | 0.145 | <.0001 |  |
|  |  | e=10 | 0.140 | <.0001 | 0.00129 | n/s | 0.144 | <.0001 |  |
|  |  | e=6 | 0.136 | <.0001 | -0.00049 | n/s | 0.139 | <.0001 |  |
|  | 6+ | perfect | 0.00623 | n/s | -0.00229 | n/s | 0.0163 | n/s |  |
|  |  | e=10 | 0.00623 | n/s | -0.00229 | n/s | 0.0163 | n/s |  |
|  |  | e=6 | 0.00384 | n/s | -0.00575 | n/s | 0.0148 | n/s |  |
|  | 14+ | perfect | n/a | n/a | n/a | n/a | n/a | n/a |  |
|  |  | e=10 | n/a | n/a | n/a | n/a | n/a | n/a |  |
|  |  | e=6 | n/a | n/a | n/a | n/a | n/a | n/a |  |
| 2 (AT) | 2 | perfect | -0.177 | <.0001 | -0.0535 | <.0001 | -0.153 | <.0001 |  |
|  |  | e=10 | -0.178 | <.0001 | -0.0544 | <.0001 | -0.154 | <.0001 |  |
|  |  | e=6 | -0.167 | <.0001 | -0.0535 | <.0001 | -0.147 | <.0001 |  |
|  | 3 to 5 | perfect | -0.115 | <.0001 | -0.0475 | 0.0003 | -0.0791 | <.0001 |  |
|  |  | e=10 | -0.116 | <.0001 | -0.0480 | 0.0003 | -0.0801 | <.0001 |  |
|  |  | e=6 | -0.149 | <.0001 | -0.0559 | <.0001 | -0.110 | <.0001 |  |
|  | 6+ | perfect | 0.0356 | 0.0074 | 0.0320 | n/s | 0.0345 | n/s |  |
|  |  | e=10 | 0.0287 | n/s | 0.0276 | n/s | 0.0313 | n/s |  |
|  |  | e=6 | 0.0007 | n/s | 0.00969 | n/s | 0.00468 | n/s |  |
|  | 10+ | perfect | 0.0225 | n/s | 0.0209 | n/s | 0.0163 | n/s |  |
|  |  | e=10 | 0.0208 | n/s | 0.0205 | n/s | 0.0163 | n/s |  |
|  |  | e=6 | 0.0208 | n/s | 0.0221 | n/s | 0.0225 | n/s |  |
| 2 (AC) | 2 | perfect | 0.0860 | <.0001 | 0.0071 | n/s | 0.0806 | <.0001 |  |
|  |  | e=10 | 0.0846 | <.0001 | 0.00668 | n/s | 0.0796 | <.0001 |  |
|  |  | e=6 | 0.0739 | <.0001 | 0.00267 | n/s | 0.0690 | <.0001 |  |
|  | 3 to 5 | perfect | 0.00766 | n/s | -0.0048 | n/s | 0.00388 | n/s |  |
|  |  | e=10 | 0.00534 | n/s | -0.00641 | n/s | 0.00366 | n/s |  |
|  |  | e=6 | 0.0403 | 0.0024 | 0.00955 | n/s | 0.0394 | 0.0061 |  |
|  | 6+ | perfect | 0.0269 | n/s | 0.0222 | n/s | 0.0214 | n/s |  |
|  |  | e=10 | 0.0318 | n/s | 0.0253 | n/s | 0.0273 | n/s |  |
|  |  | e=6 | 0.0155 | n/s | 0.00222 | n/s | 0.0111 | n/s |  |

**Table S4:** continued

| **Repeat type** | | | **RF v DSB intensity** | | **Controlling for GC** | | **Controlling for TF** | |  |
| --- | --- | --- | --- | --- | --- | --- | --- | --- | --- |
| Motif length | Repeat  count | Mismatch  type | Coeff. | P value | Coeff. | P value | Coeff. | P value | |
| 2 (AC) | 10+ | perfect | 0.0261 | n/s | 0.0235 | n/s | 0.0307 | n/s |  |
|  |  | e=10 | 0.0337 | n/s | 0.0283 | n/s | 0.0307 | n/s |  |
|  |  | e=6 | 0.0366 | 0.0058 | 0.0295 | n/s | 0.0307 | n/s |  |
| 2 (AG) | 2 | perfect | 0.0234 | n/s | -0.0158 | n/s | 0.0305 | n/s |  |
|  |  | e=10 | 0.0229 | n/s | -0.0161 | n/s | 0.0297 | n/s |  |
|  |  | e=6 | 0.0214 | n/s | -0.0184 | n/s | 0.0291 | n/s |  |
|  | 3 to 5 | perfect | -0.044 | 0.0009 | -0.0442 | 0.0009 | -0.0371 | 0.0099 |  |
|  |  | e=10 | -0.0441 | 0.0009 | -0.0441 | 0.0009 | -0.0370 | n/s |  |
|  |  | e=6 | -0.0328 | n/s | -0.0328 | n/s | -0.0312 | n/s |  |
|  | 6+ | perfect | 0.0137 | n/s | 0.0149 | n/s | n/a | n/a |  |
|  |  | e=10 | -0.00878 | n/s | -0.008 | n/s | -0.0146 | n/s |  |
|  |  | e=6 | -0.00097 | n/s | 0.00133 | n/s | 0.00535 | n/s |  |
|  | 10+ | perfect | -0.00136 | n/s | 0.00616 | n/s | n/a | n/a |  |
|  |  | e=10 | -0.01052 | n/s | -0.00688 | n/s | -0.0146 | n/s |  |
|  |  | e=6 | -0.01052 | n/s | -0.00688 | n/s | -0.0146 | n/s |  |
| 2 (CG) | 2 | perfect | 0.0616 | <.0001 | -0.0126 | n/s | 0.0613 | <.0001 |  |
|  |  | e=10 | 0.0599 | <.0001 | -0.0139 | n/s | 0.0600 | <.0001 |  |
|  |  | e=6 | 0.0559 | <.0001 | -0.0157 | n/s | 0.0575 | <.0001 |  |
|  | 3 to 5 | perfect | 0.0229 | n/s | 0.00805 | n/s | 0.0235 | n/s |  |
|  |  | e=10 | 0.0229 | n/s | 0.00805 | n/s | 0.0235 | n/s |  |
|  |  | e=6 | 0.0341 | n/s | 0.0104 | n/s | 0.0342 | n/s |  |
|  | 6+ | perfect | n/a | n/a | n/a | n/a | n/a | n/a |  |
|  |  | e=10 | n/a | n/a | n/a | n/a | n/a | n/a |  |
|  |  | e=6 | -0.00075 | n/s | -0.00825 | n/s | n/a | n/a |  |
|  | 10+ | perfect | n/a | n/a | n/a | n/a | n/a | n/a |  |
|  |  | e=10 | n/a | n/a | n/a | n/a | n/a | n/a |  |
|  |  | e=6 | n/a | n/a | n/a | n/a | n/a | n/a |  |
| 2 (all  motifs) | 2 | perfect | 0.00886 | n/s | -0.0180 | n/s | 0.0168 | n/s |  |
|  |  | e=10 | 0.00734 | n/s | -0.0192 | n/s | 0.0153 | n/s |  |
|  |  | e=6 | 0.00484 | n/s | -0.0258 | n/s | 0.0139 | n/s |  |
|  | 3 to 5 | perfect | -0.0339 | n/s | -0.0181 | n/s | -0.0244 | n/s |  |
|  |  | e=10 | -0.0355 | 0.0075 | -0.0186 | n/s | -0.0250 | n/s |  |
|  |  | e=6 | -0.0395 | 0.0029 | -0.0153 | n/s | -0.0251 | n/s |  |
|  | 6+ | perfect | 0.0424 | 0.0014 | 0.0385 | 0.0037 | 0.0350 | n/s |  |
|  |  | e=10 | 0.00886 | n/s | 0.0108 | n/s | 0.00933 | n/s |  |
|  |  | e=6 | 0.00789 | n/s | 0.00846 | n/s | 0.0132 | n/s |  |
|  | 10+ | perfect | 0.0314 | n/s | 0.0312 | n/s | 0.0341 | n/s |  |
|  |  | e=10 | 0.0295 | n/s | 0.0279 | n/s | 0.0245 | n/s |  |
|  |  | e=6 | 0.0329 | n/s | 0.0312 | n/s | 0.0295 | n/s |  |
| 3 (all  motifs) | 2 | perfect | 0.0259 | n/s | -0.00065 | n/s | 0.0181 | n/s |  |
|  |  | e=10 | 0.0257 | n/s | -0.00161 | n/s | 0.0180 | n/s |  |
|  |  | e=6 | 0.0225 | n/s | -0.00374 | n/s | 0.0160 | n/s |  |
|  | 3 to 5 | perfect | 0.0260 | n/s | 0.0192 | n/s | 0.0328 | n/s |  |
|  |  | e=10 | 0.0244 | n/s | 0.0191 | n/s | 0.0324 | n/s |  |
|  |  | e=6 | 0.0321 | n/s | 0.0207 | n/s | 0.0359 | n/s |  |

**Table S4:** continued

| **Repeat type** | | | **RF v DSB intensity** | | **Controlling for GC** | | **Controlling for TF** | |  |
| --- | --- | --- | --- | --- | --- | --- | --- | --- | --- |
| Motif length | Repeat  count | Mismatch  type | Coeff. | P value | Coeff. | P value | Coeff. | P value | |
| 3 (all | 6+ | perfect | 0.00128 | n/s | -0.00393 | n/s | 0.00875 | n/s |  |
| motifs) |  | e=10 | 0.0157 | n/s | 0.00667 | n/s | 0.0226 | n/s |  |
|  |  | e=6 | 0.0127 | n/s | 0.00481 | n/s | 0.0200 | n/s |  |
|  | 10+ | perfect | -0.0119 | n/s | -0.00959 | n/s | 0.00045 | n/s |  |
|  |  | e=10 | -0.00498 | n/s | -0.00027 | n/s | 0.00536 | n/s |  |
|  |  | e=6 | -0.0138 | n/s | -0.01411 | n/s | -0.00654 | n/s |  |
| 4 (all | 2 | perfect | -0.0400 | 0.0026 | -0.00994 | n/s | -0.0352 | n/s |  |
| motifs) |  | e=10 | -0.0397 | 0.0028 | -0.00961 | n/s | -0.0329 | n/s |  |
|  |  | e=6 | -0.0416 | 0.0017 | -0.0147 | n/s | -0.0319 | n/s |  |
|  | 3 to 5 | perfect | -0.0216 | n/s | -0.00559 | n/s | -0.02415 | n/s |  |
|  |  | e=10 | -0.0346 | 0.0091 | -0.0116 | n/s | -0.0388 | 0.0069 |  |
|  |  | e=6 | -0.0432 | 0.0011 | -0.0179 | n/s | -0.0419 | 0.0035 |  |
|  | 6+ | perfect | 0.0191 | n/s | 0.0138 | n/s | 0.0211 | n/s |  |
|  |  | e=10 | 0.0191 | n/s | 0.0138 | n/s | 0.0211 | n/s |  |
|  |  | e=6 | 0.0118 | n/s | 0.00536 | n/s | 0.0113 | n/s |  |
|  | 10+ | perfect | n/a | n/a | n/a | n/a | n/a | n/a |  |
|  |  | e=10 | n/a | n/a | n/a | n/a | n/a | n/a |  |
|  |  | e=6 | n/a | n/a | n/a | n/a | n/a | n/a |  |
| 5 (all | 2 | perfect | -0.0568 | <.0001 | -0.0226 | n/s | -0.0417 | 0.0037 |  |
| motifs) |  | e=10 | -0.0622 | <.0001 | -0.0266 | n/s | -0.0469 | 0.0011 |  |
|  |  | e=6 | -0.0572 | <.0001 | -0.0266 | n/s | -0.0419 | 0.0035 |  |
|  | 3 to 5 | perfect | 0.00996 | n/s | 0.0112 | n/s | 0.00911 | n/s |  |
|  |  | e=10 | 0.00372 | n/s | 0.0198 | n/s | 0.0124 | n/s |  |
|  |  | e=6 | 0.00467 | n/s | 0.0202 | n/s | 0.0128 | n/s |  |
|  | 6+ | perfect | n/a | n/a | n/a | n/a | n/a | n/a |  |
|  |  | e=10 | 0.0116 | n/s | 0.00674 | n/s | -0.0124 | n/s |  |
|  |  | e=6 | 0.0192 | n/s | 0.0126 | n/s | 0.00402 | n/s |  |
|  | 10+ | perfect | n/a | n/a | n/a | n/a | n/a | n/a |  |
|  |  | e=10 | n/a | n/a | n/a | n/a | n/a | n/a |  |
|  |  | e=6 | 0.01592 | n/s | 0.0093 | n/s | n/a | n/a |  |
| 6 (all | 2 | perfect | -0.00926 | n/s | -0.00789 | n/s | -0.0150 | n/s |  |
| motifs) |  | e=10 | -0.0104 | n/s | -0.00814 | n/s | -0.0152 | n/s |  |
|  |  | e=6 | -0.0162 | n/s | -0.0137 | n/s | -0.0240 | n/s |  |
|  | 3 to 5 | perfect | 0.0330 | n/s | 0.0195 | n/s | 0.0169 | n/s |  |
|  |  | e=10 | 0.0294 | n/s | 0.0143 | n/s | 0.0111 | n/s |  |
|  |  | e=6 | 0.0282 | n/s | 0.0175 | n/s | 0.0158 | n/s |  |
|  | 6+ | perfect | 0.00192 | n/s | 0.00066 | n/s | 0.00417 | n/s |  |
|  |  | e=10 | 0.0217 | n/s | 0.0117 | n/s | 0.0243 | n/s |  |
|  |  | e=6 | 0.0326 | n/s | 0.0210 | n/s | 0.0406 | 0.0047 |  |
|  | 10+ | perfect | n/a | n/a | n/a | n/a | n/a | n/a |  |
|  |  | e=10 | 0.0232 | n/s | 0.0195 | n/s | 0.0268 | n/s |  |
|  |  | e=6 | 0.0181 | n/s | 0.0107 | n/s | 0.0234 | n/s |  |

**Table S5:** Mean GC content of microsatellites with at least six copies for all IGRs in the *S. cerevisiae* genome. IGRs were divided by recombination (double-strand break) intensity as reported by Gerton and co-workers [1] into 473 hot, 89 cold and 5431 other regions, which were all IGRs not categorized as either hot or cold. The e value denotes the number of bases in any part of a repeat within which no more than one mismatch was allowed with respect to the consensus repeated motif.

| Repeat type | | **Mean repeat GC content (6-copy repeats and longer) and total number of repeats by IGR type** | | | | | | **P value** | |
| --- | --- | --- | --- | --- | --- | --- | --- | --- | --- |
| Motif  length | Mismatch  type | Hot | | Other | | Cold | | Hot v  non-hot | Cold v other |
|  |  | Mean | n | Mean | n | Mean | n |  |  |
| 3 (all  motifs) | perfect | 0 | 7 | 0.148148 | 27 | n/a | 0 | n/s | n/a |
|  | e=10 | 0.094418 | 11 | 0.191032 | 66 | n/a | 0 | n/s | n/a |
|  | e=6 | 0.192279 | 21 | 0.169623 | 118 | n/a | 0 | n/s | n/a |
| 4 (all  motifs) | perfect | n/a | 0 | 0.05 | 5 | n/a | 0 | n/a | n/a |
|  | e=10 | n/a | 0 | 0.082383 | 12 | n/a | 0 | n/a | n/a |
|  | e=6 | n/a | 0 | 0.1095 | 19 | n/a | 0 | n/a | n/a |
| 5 (all  motifs) | perfect | n/a | 0 | 0.4 | 2 | n/a | 0 | n/a | n/a |
|  | e=10 | 0.457143 | 1 | 0.508084 | 4 | n/a | 0 | n/a | n/a |
|  | e=6 | 0.457143 | 1 | 0.221561 | 5 | n/a | 0 | n/a | n/a |
| 6 (all  motifs) | perfect | 0.166667 | 1 | 0.5 | 3 | n/a | 0 | n/s | n/a |
|  | e=10 | 0.189189 | 1 | 0.582525 | 21 | n/a | 0 | n/s | n/a |
|  | e=6 | 0.189189 | 1 | 0.360875 | 10 | n/a | 0 | n/s | n/a |

**Table S6:** Mean GC content of microsatellites with at least six copies for all ORFs in the *S. cerevisiae* genome. ORFs were divided by recombination (double-strand break) intensity as reported by Gerton and co-workers [1] into 297 hot, 49 cold and 5634 other regions, which were all ORFs not categorized as either hot or cold. The e value denotes the number of bases in any part of a repeat within which no more than one mismatch was allowed with respect to the consensus repeated motif.

| Repeat type | | **Mean repeat GC content (6-copy repeats and longer) and total number of repeats by ORF type** | | | | | | **P value** | |
| --- | --- | --- | --- | --- | --- | --- | --- | --- | --- |
| Motif  length | Mismatch  type | Hot | | Other | | Cold | | Hot v  non-hot | Cold v other |
|  |  | Mean | n | Mean | n | Mean | n |  |  |
| 3 (all | perfect | 0.416666 | 8 | 0.353535 | 165 | n/a | 0 | n/s | n/a |
| motifs) | e=10 | 0.352323 | 20 | 0.36545 | 316 | n/a | 0 | n/s | n/a |
|  | e=6 | 0.405133 | 28 | 0.387144 | 474 | 0.42862 | 4 | n/s | n/s |
| 4 (all | perfect | n/a | 0 | 0.25 | 1 | n/a | 0 | n/a | n/a |
| motifs) | e=10 | n/a | 0 | 0.25 | 1 | n/a | 0 | n/a | n/a |
|  | e=6 | n/a | 0 | 0.280173 | 2 | n/a | 0 | n/a | n/a |
| 5 (all | perfect | n/a | 0 | n/a | 0 | n/a | 0 | n/a | n/a |
| motifs) | e=10 | n/a | 0 | 0.298019 | 3 | n/a | 0 | n/a | n/a |
|  | e=6 | n/a | 0 | 0.232278 | 4 | n/a | 0 | n/a | n/a |
| 6 (all | perfect | n/a | 0 | 0.388889 | 3 | n/a | 0 | n/a | n/a |
| motifs) | e=10 | 0.413691 | 2 | 0.487103 | 15 | n/a | 0 | n/s | n/a |
|  | e=6 | 0.466199 | 4 | 0.459199 | 11 | n/a | 0 | n/s | n/a |

**Table S7:** Mean lengths of microsatellites of at least six copies for IGRs throughout the yeast genome. IGRs were divided by recombination (double-strand break) intensity as reported by Gerton and co-workers [1] into 473 hot, 89 cold and 5431 other regions, which were all IGRs not categorized as either hot or cold. The e value denotes the number of bases in any part of a repeat within which no more than one mismatch was allowed with respect to the consensus repeated motif. All p values <0.01 are shown, but caution is recommended in view of the multiple hypotheses being tested.

| **Repeat type** | | **Mean repeat length (6-copy repeats and longer)**  **And total number of repeats by IGR type** | | | | | | **P value** | |
| --- | --- | --- | --- | --- | --- | --- | --- | --- | --- |
| Motif  length | Mismatch  type | Hot | | Other | | Cold | | Hot v non-  hot | Cold v  other |
|  |  | Mean | N | mean | N | mean | N |  |  |
| 1 (A) | perfect | 8.24 | 1174 | 7.63 | 11388 | 7.19 | 240 | < 0.0001 | n/s |
|  | e=10 | 8.67 | 1236 | 7.87 | 12025 | 7.37 | 237 | < 0.0001 | n/s |
|  | e=6 | 9.26 | 1473 | 8.53 | 14870 | 8.05 | 294 | < 0.0001 | n/s |
| 1 (G) | perfect | 6.52 | 31 | 6.37 | 232 | 6.43 | 7 | n/s | n/s |
|  | e=10 | 7.16 | 32 | 6.44 | 233 | 6.43 | 7 | 0.0059 | n/s |
|  | e=6 | 8.09 | 46 | 7.13 | 298 | 7.00 | 7 | 0.0038 | n/s |
| 2 (AT) | perfect | 8.70 | 46 | 8.89 | 308 | 8.50 | 2 | n/s | n/s |
|  | e=10 | 9.65 | 66 | 9.74 | 429 | 8.13 | 4 | n/s | n/s |
|  | e=6 | 8.80 | 99 | 8.41 | 836 | 7.25 | 8 | n/s | n/s |
| 2 (AC) | perfect | 8.13 | 8 | 9.19 | 37 | n/a | 0 | n/s | n/a |
|  | e=10 | 8.59 | 11 | 9.13 | 47 | n/a | 0 | n/s | n/a |
|  | e=6 | 7.73 | 22 | 15.93 | 108 | 6.25 | 2 | n/s | n/s |
| 2 (AG) | perfect | 6.67 | 3 | 10.67 | 9 | 8 | 1 | n/s | n/s |
|  | e=10 | 8.70 | 5 | 8.32 | 25 |  | 0 | n/s | n/s |
|  | e=6 | 7.50 | 9 | 7.24 | 63 | 6.75 | 4 | n/s | n/s |
| 2 (CG) | e=10 | n/a | 0 | 6.5 | 1 | n/a | 0 | n/a | n/a |
|  | e=6 | n/a | 0 | 6.5 | 1 | n/a | 0 | n/a | n/a |
| 2 (all  motifs) | perfect | 8.51 | 57 | 8.96 | 354 | 8.33 | 3 | n/s | n/s |
|  | e=10 | 9.45 | 82 | 9.60 | 502 | 8.30 | 5 | n/s | n/s |
|  | e=6 | 8.53 | 130 | 9.14 | 1008 | 6.96 | 14 | n/s | n/s |
| 3 (all  motifs) | perfect | 7.86 | 7 | 9.89 | 27 | n/a | 0 | n/s | n/a |
|  | e=10 | 9.24 | 11 | 9.36 | 66 | n/a | 0 | n/s | n/a |
|  | e=6 | 8.30 | 21 | 8.41 | 118 | n/a | 0 | n/s | n/a |
| 4 (all  motifs) | perfect | n/a | 0 | 7.80 | 5 | n/a | 0 | n/a | n/a |
|  | e=10 | n/a | 0 | 8.00 | 12 | n/a | 0 | n/a | n/a |
|  | e=6 | n/a | 0 | 7.88 | 19 | n/a | 0 | n/a | n/a |
| 5 (all  motifs) | perfect | n/a | 0 | 6.50 | 2 | n/a | 0 | n/a | n/a |
|  | e=10 | 6.6 | 1 | 6.95 | 4 | n/a | 0 | n/a | n/a |
|  | e=6 | 6.6 | 1 | 6.80 | 5 | n/a | 0 | n/a | n/a |
| 6 (all  motifs) | perfect | 6 | 1 | 7.00 | 3 | n/a | 0 | n/s | n/a |
|  | e=10 | 6 | 1 | 9.65 | 21 | n/a | 0 | n/s | n/a |
|  | e=6 | 6 | 1 | 9.67 | 10 | n/a | 0 | n/s | n/a |

**Table S8:** Mean lengths of microsatellites of at least six copies for ORFs throughout the yeast genome.ORFs were divided by recombination (double-strand break) intensity as reported by Gerton and co-workers [1] into 297 hot, 49 cold and 5634 other regions, which were all ORFs not categorized as either hot or cold. The e value denotes the number of bases in any part of a repeat within which no more than one mismatch was allowed with respect to the consensus repeated motif. All p values <0.01 are shown, but caution is recommended in view of the multiple hypotheses being tested.

| **Repeat type** | | **Mean repeat length (6-copy repeats and longer)**  **and total number of repeats by ORF type** | | | | | | **P value** | |
| --- | --- | --- | --- | --- | --- | --- | --- | --- | --- |
| Motif  length | Mismatch  type | Hot | | Other | | Cold | | Hot v non-  hot | Cold v  other |
|  |  | mean | N | mean | N | mean | N |  |  |
| 1 (A) | perfect | 6.70 | 315 | 6.53 | 12329 | 6.43 | 151 | n/s | n/s |
|  | e=10 | 6.73 | 338 | 6.57 | 13344 | 6.47 | 151 | n/s | n/s |
|  | e=6 | 7.31 | 436 | 7.25 | 17469 | 7.10 | 186 | n/s | n/s |
| 1 (G) | perfect | 6.19 | 32 | 6.21 | 466 | 6.13 | 8 | n/s | n/s |
|  | e=10 | 6.19 | 32 | 6.22 | 466 | 6.13 | 8 | n/s | n/s |
|  | e=6 | 6.86 | 44 | 6.70 | 632 | 6.50 | 10 | n/s | n/s |
| 2 (AT) | perfect | 7.83 | 6 | 8.20 | 15 | n/a | 0 | n/s | n/a |
|  | e=10 | 9.94 | 8 | 8.77 | 30 | n/a | 0 | n/s | n/a |
|  | e=6 | 9.36 | 11 | 7.26 | 88 | n/a | 0 | n/s | n/a |
| 2 (AC) | perfect | 19 | 1 | 7.60 | 5 | n/a | 0 | n/s | n/a |
|  | e=10 | 10.8 | 3 | 9.11 | 9 | n/a | 0 | n/s | n/a |
|  | e=6 | 8.43 | 7 | 8.59 | 59 | n/a | 0 | n/s | n/a |
| 2 (AG) | perfect | 7 | 1 | 10 | 1 | n/a | 0 | n/s | n/a |
|  | e=10 | 8.5 | 1 | 7.11 | 14 | n/a | 0 | n/s | n/a |
|  | e=6 | 7.08 | 6 | 6.48 | 97 | n/a | 0 | n/s | n/a |
| 2 (CG) | e=10 | n/a | 0 | n/a | 0 | n/a | 0 | n/a | n/a |
|  | e=6 | n/a | 0 | 6 | 1 | n/a | 0 | n/a | n/a |
| 2 (all  motifs) | perfect | 9.13 | 8 | 8.14 | 21 | n/a | 0 | n/s | n/a |
|  | e=10 | 10.0 | 12 | 8.39 | 53 | n/a | 0 | n/s | n/a |
|  | e=6 | 8.52 | 24 | 7.27 | 245 | n/a | 0 | 0.00185 | n/a |
| 3 (all  motifs) | perfect | 6.88 | 8 | 8.07 | 165 | n/a | 0 | n/s | n/a |
|  | e=10 | 7.35 | 20 | 8.71 | 316 | n/a | 0 | n/s | n/a |
|  | e=6 | 7.35 | 28 | 8.67 | 474 | 6.92 | 4 | n/s | n/s |
| 4 (all  motifs) | perfect | n/a | 0 | 6 | 1 | n/a | 0 | n/a | n/a |
|  | e=10 | n/a | 0 | 6 | 1 | n/a | 0 | n/a | n/a |
|  | e=6 | n/a | 0 | 6.25 | 2 | n/a | 0 | n/a | n/a |
| 5 (all  motifs) | perfect | n/a | 0 | n/a | 0 | n/a | 0 | n/a | n/a |
|  | e=10 | n/a | 0 | 7.2 | 3 | n/a | 0 | n/a | n/a |
|  | e=6 | n/a | 0 | 9.1 | 4 | n/a | 0 | n/a | n/a |
| 6 (all  motifs) | perfect | n/a | 0 | 6 | 3 | n/a | 0 | n/a | n/a |
|  | e=10 | 8.92 | 2 | 7.41 | 15 | n/a | 0 | n/s | n/a |
|  | e=6 | 7.96 | 4 | 10.0 | 11 | n/a | 0 | n/s | n/a |

**Table S9:** The five most common multiply represented trinucleotide repeat motifs for each type of region. Poly purine/poly pyrimidine motifs are emboldened. Perfect repeats only were considered for this analysis.

| **2 copy repeats** | | | **3 to 5 copy repeats** | | | **6+ copy repeats** | | | | |
| --- | --- | --- | --- | --- | --- | --- | --- | --- | --- | --- |
| Region type | Motif | N | Region type | Motif | N | Region type | | Motif | | N |
| hot ORFs | TTC | 170 | hot ORFs | TCT | 14 | hot ORFs |  | TTG | | 2 |
|  | **GAA** | 169 |  | **AAG** | 13 |  |  |  |  |  |
|  | CAA | 156 |  | AGC | 13 |  |  |  |  |  |
|  | CTT | 150 |  | GAA | 13 |  |  |  |  |  |
|  | TTG | 146 |  | AAC | 12 |  |  |  |  |  |
|  |  |  |  | AGA | 12 |  |  |  |  |  |
|  |  |  |  | TCA | 12 |  |  |  |  |  |
| hot IGRs | AAT | 139 | hot IGRs | TAT | 15 | hot IGRs |  | TAT | | 4 |
|  | TAT | 131 |  | ATA | 11 |  |  | AAT | | 1 |
|  | AAG | 124 |  | ATT | 10 |  |  | ATT | | 1 |
|  | **TTC** | 123 |  | AAT | 9 |  |  | TTA | | 1 |
|  | **GAA** | 122 |  | TTC | 9 |  |  |  |  |  |
| other ORFs | TTC | 4787 | other ORFs | TTC | 353 | other ORFs |  | CAG | | 12 |
|  | **GAA** | 4778 |  | **GAA** | 351 |  |  | TCA | | 12 |
|  | **AAG** | 4017 |  | TCA | 264 |  |  | TTC | | 12 |
|  | AAT | 3992 |  | AAG | 256 |  |  | CAA | | 10 |
|  | ATT | 3743 |  | **TCT** | 251 |  |  | TGT | | 10 |
| other IGRs | AAT | 1896 | other IGRs | TAT | 153 | other IGRs |  | TAT | | 6 |
|  | TAT | 1697 |  | ATA | 138 |  |  | ATA | | 3 |
|  | ATT | 1604 |  | AAT | 127 |  |  | TAA | | 3 |
|  | ATA | 1562 |  | TAA | 110 |  |  | AAT | | 2 |
|  | TTA | 1408 |  | TTA | 100 |  |  | CAA | | 2 |
|  |  |  |  |  |  |  |  | GAA | | 2 |
|  |  |  |  |  |  |  |  | TAG | | 2 |
| cold ORFs | ATT | 47 | cold ORFs | TGC | 6 |  |  |  |  |  |
|  | TTC | 47 |  | GAT | 5 |  |  |  |  |  |
|  | TTA | 43 |  | TCT | 5 |  |  |  |  |  |
|  | TGA | 42 |  | **AAG** | 4 |  |  |  |  |  |
|  | TCT | 41 |  | **GAA** | 4 |  |  |  |  |  |
|  |  |  |  | TTG | 4 |  |  |  |  |  |
| cold IGRs | ATT | 48 | cold IGRs | TAT | 4 |  |  |  |  |  |
|  | AAT | 46 |  | TAA | 3 |  |  |  |  |  |
|  | TTC | 38 |  | ATA | 3 |  |  |  |  |  |
|  | TTA | 35 |  | ATT | 2 |  |  |  |  |  |
|  | AAG | 33 |  | GAA | 2 |  |  |  |  |  |

**Table S10:** The five most common multiply represented tetranucleotide repeat motifs for each type of region.. Poly purine/poly pyrimidine motifs are emboldened. Perfect repeats only were considered for this analysis

| **2 copy repeats** | | | **3 to 5 copy repeats** | | | | **6+ copy repeats** | | | |
| --- | --- | --- | --- | --- | --- | --- | --- | --- | --- | --- |
| Region type | Motif | N | Region type | Motif | | N | Region type | Motif | | N |
| hot ORFs | AAAG | 19 |  |  |  |  |  |  |  |  |
|  | **TCTT** | 16 |  |  |  |  |  |  |  |  |
|  | ATTT | 15 |  |  |  |  |  |  |  |  |
|  | TTTG | 15 |  |  |  |  |  |  |  |  |
|  | TTGT | 14 |  |  |  |  |  |  |  |  |
| hot IGRs | TTTC | 31 | hot IGRs | AAAT | | 4 |  |  |  |  |
|  | **AAAG** | 30 |  | TTTC | | 4 |  |  |  |  |
|  | **GAAA** | 27 |  | **AAGA** | | 3 |  |  |  |  |
|  | TATG | 23 |  | ATAC | | 2 |  |  |  |  |
|  | AAAT | 21 |  | ATTT | | 2 |  |  |  |  |
|  | CTTT | 21 |  | GTAT | | 2 |  |  |  |  |
|  | TTAT | 21 |  | TTTA | | 2 |  |  |  |  |
| other ORFs | TTTC | 506 | other ORFs | CTTT | | 11 |  |  |  |  |
|  | **AAAG** | 445 |  | **AAAG** | | 9 |  |  |  |  |
|  | **AAGA** | 416 |  | **TTTC** | | 8 |  |  |  |  |
|  | **GAAA** | 407 |  | **AGAA** | | 6 |  |  |  |  |
|  | AAAT | 385 |  | **GAAA** | | 6 |  |  |  |  |
| other IGRs | TTTC | 308 | other IGRs | TTTA | | 22 | other IGRs | AATA |  | 2 |
|  | AAAT | 282 |  | ATAA | | 16 |  |  |  |  |
|  | AAAG | 279 |  | AAAT | | 15 |  |  |  |  |
|  | TTTA | 272 |  | AATA | | 15 |  |  |  |  |
|  | TATT | 270 |  | TATT | | 12 |  |  |  |  |
| cold ORFs | AGAA | 9 |  |  |  |  |  |  |  |  |
|  | TTTG | 8 |  |  |  |  |  |  |  |  |
|  | TCTT | 7 |  |  |  |  |  |  |  |  |
|  | **AAAG** | 6 |  |  |  |  |  |  |  |  |
|  | **TTTC** | 5 |  |  |  |  |  |  |  |  |
| cold IGRs | AAAT | 8 | cold IGRs | ATAA | | 2 |  |  |  |  |
|  | ATTT | 8 |  |  |  |  |  |  |  |  |
|  | TTAT | 8 |  |  |  |  |  |  |  |  |
|  | AAAG | 7 |  |  |  |  |  |  |  |  |
|  | AATA | 7 |  |  |  |  |  |  |  |  |

**Table S11:** The five most common multiply represented pentanucleotide repeat motifs for each type of region. Poly purine/poly pyrimidine motifs are emboldened. Perfect repeats only were considered for this analysis.

| **2 copy repeats** | | | **3 to 5 copy repeats** | | | **6+ copy repeats** | | |  |
| --- | --- | --- | --- | --- | --- | --- | --- | --- | --- |
| Region type | Motif | N | Region type | Motif | N | Region type | Motif | N |  |
| hot ORFs | GAAAA | 6 |  |  |  |  |  |  |  |
|  | **TTCTT** | 4 |  |  |  |  |  |  |  |
|  | **AAAGA** | 3 |  |  |  |  |  |  |  |
|  | **AAGAA** | 3 |  |  |  |  |  |  |  |
|  | AAGCA | 3 |  |  |  |  |  |  |  |
|  | CAGAG | 3 |  |  |  |  |  |  |  |
|  | CATTC | 3 |  |  |  |  |  |  |  |
|  | TCTTC | 3 |  |  |  |  |  |  |  |
| hot IGRs | TTTTC | 15 |  |  |  |  |  |  |  |
|  | **AGAAA** | 10 |  |  |  |  |  |  |  |
|  | **GAAAA** | 10 |  |  |  |  |  |  |  |
|  | **AAAAG** | 8 |  |  |  |  |  |  |  |
|  | AAAAT | 7 |  |  |  |  |  |  |  |
|  | TTTCT | 7 |  |  |  |  |  |  |  |
| other ORFs | TTTTC | 104 | other ORFs | CTTTT | 3 |  |  |  |  |
|  | **AAGAA** | 84 |  | GGTGT | 2 |  |  |  |  |
|  | **TTCTT** | 84 |  | TTTGT | 2 |  |  |  |  |
|  | **GAAAA** | 82 |  |  |  |  |  |  |  |
|  | AAAAT | 79 |  |  |  |  |  |  |  |
| other IGRs | TTTTC | 91 | other IGRs | CACAC | 6 |  |  |  |  |
|  | **AAAAG** | 86 |  | GAAAA | 5 |  |  |  |  |
|  | AAAAT | 82 |  | GATGA | 5 |  |  |  |  |
|  | ATATA | 58 |  | ATAAT | 4 |  |  |  |  |
|  | AAGAA | 57 |  | GGTGT | 4 |  |  |  |  |
|  | ATTTT | 57 |  | TTTTC | 4 |  |  |  |  |
| cold ORFs | TTTCT | 3 |  |  |  |  |  |  |  |
|  | ACCAA | 2 |  |  |  |  |  |  |  |
|  | AGAAT | 2 |  |  |  |  |  |  |  |
|  | GAAAA | 2 |  |  |  |  |  |  |  |
|  | TCAAA | 2 |  |  |  |  |  |  |  |
|  | TGAAT | 2 |  |  |  |  |  |  |  |
|  | TTCTG | 2 |  |  |  |  |  |  |  |
|  | TTTCC | 2 |  |  |  |  |  |  |  |
|  | **TTTTC** | 2 |  |  |  |  |  |  |  |
|  | TTTTG | 2 |  |  |  |  |  |  |  |
| cold IGRs | ATTTT | 3 |  |  |  |  |  |  |  |
|  | AAAAG | 2 |  |  |  |  |  |  |  |
|  | **AAAGG** | 2 |  |  |  |  |  |  |  |
|  | ATAAA | 2 |  |  |  |  |  |  |  |
|  | ATCTT | 2 |  |  |  |  |  |  |  |
|  | CTAAA | 2 |  |  |  |  |  |  |  |
|  | CTTTT | 2 |  |  |  |  |  |  |  |
| **Table S11:** continued | |  |  |  |  |  |  |  |  |
| **2 copy repeats** | Motif | N |  |  |  |  |  |  |  |
| Region type | Motif | N |  |  |  |  |  |  |  |
|  | TTATA | 2 |  |  |  |  |  |  |  |
|  | TTTTG | 2 |  |  |  |  |  |  |  |

**Table S12:** The five most common multiply represented hexanucleotide repeat motifs for each type of region. Poly purine/poly pyrimidine motifs are emboldened. Perfect repeats only were considered for this analysis

| 2 copy repeats | | | **3 to 5 copy repeats** | | | **6+ copy repeats** | | |
| --- | --- | --- | --- | --- | --- | --- | --- | --- |
| Region type | Motif | N | Region type | Motif | N | Region type | Motif | N |
| hot ORFs | ACCACT | 3 |  |  |  |  |  |  |
|  | CAACAG | 3 |  |  |  |  |  |  |
|  | GAAGAT | 3 |  |  |  |  |  |  |
|  | CTTTTT | 2 |  |  |  |  |  |  |
|  | GACGAA | 2 |  |  |  |  |  |  |
|  | TATACA | 2 |  |  |  |  |  |  |
|  | TCTTCG | 2 |  |  |  |  |  |  |
|  | TTCAGT | 2 |  |  |  |  |  |  |
|  | TTCGTC | 2 |  |  |  |  |  |  |
| hot IGRs | AAGAAA | 5 |  |  |  |  |  |  |
|  | AGAAAA | 4 |  |  |  |  |  |  |
|  | TATACA | 4 |  |  |  |  |  |  |
|  | TTTTTC | 4 |  |  |  |  |  |  |
|  | AAAAGA | 3 |  |  |  |  |  |  |
|  | TTTCTT | 3 |  |  |  |  |  |  |
|  | TTTTCT | 3 |  |  |  |  |  |  |
|  | TTTTTA | 3 |  |  |  |  |  |  |
| other ORFs | AGATGA | 31 | other ORFs | CAGCAA | 6 |  |  |  |
|  | AAAGAA | 26 |  | TGTTGC | 5 |  |  |  |
|  | TTCATC | 24 |  | GTTGCT | 4 |  |  |  |
|  | AAGAAA | 22 |  | TGCTGT | 4 |  |  |  |
|  | **TTTTTC** | 21 |  | GATGAA | 3 |  |  |  |
|  |  |  |  | TGTGCT | 3 |  |  |  |
| other IGRs | TTTTTC | 34 | other IGRs | CCACAC | 14 | other IGRs | GGTGTG | 2 |
|  | GTGTGG | 33 |  | GTGTGG | 10 |  |  |  |
|  | CCACAC | 30 |  | AAAACA | 3 |  |  |  |
|  | TTTCTT | 30 |  | AAAGAA | 3 |  |  |  |
|  | **AAAAAG** | 29 |  | CTTTTT | 2 |  |  |  |
|  |  |  |  | GCGGAA | 2 |  |  |  |
|  |  |  |  | GGTGTG | 2 |  |  |  |
|  |  |  |  | TATATG | 2 |  |  |  |
| cold ORFs | ACCGAG | 5 |  |  |  |  |  |  |
| cold IGRs | GAAAAA | 2 |  |  |  |  |  |  |
|  | TGTTTT | 2 |  |  |  |  |  |  |

**Table S13:** Mean per kb frequencies of short tandem repeats for all IGRs in the *S. cerevisiae* genome divided according to the number of promoters they contain into 1537 with no promoters, 2894 with one and 1530 with two. P values are for Kruskal Wallis non- parametric ANOVA. The e value denotes the number of bases in any part of a repeat within which no more than one mismatch was allowed with respect to the consensus repeated motif. All p values <0.01 are shown, but caution is recommended in view of the multiple hypotheses being tested.

| **Repeat type** | | | **Mean per kb repeat**  **frequencies for hot IGRs** | | | | **Mean per kb repeat**  **frequencies for non-hot IGRs** | | | |
| --- | --- | --- | --- | --- | --- | --- | --- | --- | --- | --- |
| Motif  length | Copy  number | Mismatch  type | Number of promoters | | | P value | Number of promoters | | | P value |
|  |  |  | None | One | Two |  | None | One | Two |  |
| 1 (A) | 3 to 5 | perfect | 35.0 | 35.5 | 34.2 | n/s | 41.0 | 39.8 | 39.1 | < 0.0001 |
|  |  | e=10 | 33.6 | 34.9 | 33.8 | n/s | 40.5 | 39.4 | 38.7 | 0.00028 |
|  |  | e=6 | 30.9 | 32.3 | 31.6 | n/s | 37.1 | 36.7 | 36.3 | n/s |
|  | 6+ | perfect | 6.71 | 5.51 | 4.36 | 0.00289 | 5.12 | 4.61 | 4.15 | 0.00037 |
|  |  | e=10 | 6.49 | 5.33 | 4.21 | 0.00371 | 4.99 | 4.49 | 4.07 | 0.00144 |
|  |  | e=6 | 7.96 | 6.04 | 4.98 | 0.00037 | 6.09 | 5.53 | 5.01 | < 0.0001 |
|  | 14+ | perfect | 0.503 | 0.473 | 0.261 | n/s | 0.226 | 0.174 | 0.111 | n/s |
|  |  | e=10 | 1.08 | 0.692 | 0.564 | n/s | 0.437 | 0.294 | 0.220 | n/s |
|  |  | e=6 | 1.25 | 0.821 | 0.632 | n/s | 0.495 | 0.370 | 0.277 | n/s |
| 1 (G) | 3 to 5 | perfect | 6.62 | 9.71 | 10.0 | < 0.0001 | 5.24 | 7.58 | 8.42 | < 0.0001 |
|  |  | e=10 | 6.62 | 9.68 | 10.0 | < 0.0001 | 5.22 | 7.57 | 8.43 | < 0.0001 |
|  |  | e=6 | 6.36 | 9.40 | 9.76 | < 0.0001 | 5.16 | 7.44 | 8.29 | < 0.0001 |
|  | 6+ | perfect | 0.0788 | 0.133 | 0.117 | n/s | 0.0618 | 0.0744 | 0.0904 | < 0.0001 |
|  |  | e=10 | 0.0788 | 0.133 | 0.103 | n/s | 0.0571 | 0.0744 | 0.0904 | < 0.0001 |
|  |  | e=6 | 0.156 | 0.175 | 0.138 | n/s | 0.0652 | 0.0959 | 0.119 | < 0.0001 |
|  | 14+ | perfect | 0 | 0 | 0.0121 | n/s | 0 | 0.00151 | 0 | n/s |
|  |  | e=10 | 0 | 0 | 0.0121 | n/s | 0 | 0.00151 | 0 | n/s |
|  |  | e=6 | 0 | 0 | 0.0121 | n/s | 0 | 0.00151 | 0 | n/s |
| 2 (AT) | 2 | perfect | 9.80 | 7.27 | 6.94 | n/s | 11.3 | 9.02 | 7.64 | < 0.0001 |
|  |  | e=10 | 9.46 | 7.22 | 6.85 | n/s | 11.0 | 8.91 | 7.58 | < 0.0001 |
|  |  | e=6 | 7.14 | 6.16 | 5.83 | n/s | 8.82 | 7.50 | 6.59 | < 0.0001 |
|  | 3 to 5 | perfect | 4.30 | 2.48 | 1.91 | n/s | 4.21 | 2.41 | 1.47 | < 0.0001 |
|  |  | e=10 | 3.87 | 2.22 | 1.76 | n/s | 3.90 | 2.29 | 1.39 | < 0.0001 |
|  |  | e=6 | 4.60 | 2.75 | 2.41 | n/s | 4.94 | 3.07 | 1.98 | < 0.0001 |
|  | 6+ | perfect | 0.882 | 0.153 | 0.177 | n/s | 0.377 | 0.137 | 0.0478 | < 0.0001 |
|  |  | e=10 | 1.11 | 0.286 | 0.276 | n/s | 0.497 | 0.183 | 0.0871 | < 0.0001 |
|  |  | e=6 | 1.45 | 0.485 | 0.300 | n/s | 0.956 | 0.352 | 0.145 | < 0.0001 |
|  | 10+ | perfect | 0.546 | 0.0378 | 0.0430 | n/s | 0.137 | 0.0391 | 0.00826 | 0.000273 |
|  |  | e=10 | 0.678 | 0.0639 | 0.0915 | n/s | 0.208 | 0.0591 | 0.0224 | 0.000116 |
|  |  | e=6 | 0.684 | 0.0879 | 0.129 | n/s | 0.222 | 0.0737 | 0.0260 | 0.001032 |
| 2 (AC) | 2 | perfect | 6.14 | 6.81 | 7.26 | n/s | 6.33 | 6.60 | 6.86 | < 0.0001 |
|  |  | e=10 | 5.92 | 6.69 | 7.21 | n/s | 6.28 | 6.57 | 6.84 | < 0.0001 |
|  |  | e=6 | 5.46 | 6.04 | 6.78 | n/s | 5.80 | 6.03 | 6.22 | < 0.0001 |
|  | 3 to 5 | perfect | 0.964 | 0.922 | 0.844 | n/s | 0.574 | 0.573 | 0.611 | < 0.0001 |
|  |  | e=10 | 1.03 | 0.926 | 0.844 | n/s | 0.559 | 0.563 | 0.603 | < 0.0001 |
|  |  | e=6 | 1.45 | 1.31 | 1.27 | n/s | 0.934 | 0.966 | 1.06 | < 0.0001 |
|  | 6+ | perfect | 0.0755 | 0.0461 | 0.0452 | n/s | 0.0183 | 0.0138 | 0.00924 | n/s |
|  |  | e=10 | 0.151 | 0.0659 | 0.0452 | n/s | 0.0276 | 0.0210 | 0.0118 | n/s |
|  |  | e=6 | 0.332 | 0.0937 | 0.0671 | n/s | 0.0460 | 0.0387 | 0.0400 | n/s |

### Table S13: continued

| **Repeat type** | | | **Mean per kb repeat**  **frequencies for hot IGRs** | | | | **Mean per kb repeat**  **frequencies for non-hot IGRs** | | | |
| --- | --- | --- | --- | --- | --- | --- | --- | --- | --- | --- |
| Motif  length | Copy  number | Mismatch  type | Number of promoters | | | P value | Number of promoters | | | P value |
|  |  |  | None | One | Two |  | None | One | Two |  |
| 2 (AC) | 10+ | perfect | 0.0755 | 0 | 0.00203 | n/s | 0.00458 | 0.00401 | 0.00083 | n/s |
|  |  | e=10 | 0.0755 | 0.0117 | 0.00203 | n/s | 0.00574 | 0.00401 | 0.00083 | n/s |
|  |  | e=6 | 0.0755 | 0.0244 | 0.00203 | n/s | 0.00402 | 0.00415 | 0.00179 | n/s |
| 2 (AG) | 2 | perfect | 6.78 | 7.65 | 7.98 | 0.00116 | 5.98 | 7.04 | 8.12 | < 0.0001 |
|  |  | e=10 | 6.81 | 7.57 | 7.97 | 0.00221 | 5.97 | 7.02 | 8.08 | < 0.0001 |
|  |  | e=6 | 6.23 | 6.71 | 7.13 | 0.00479 | 5.44 | 6.31 | 7.30 | < 0.0001 |
|  | 3 to 5 | perfect | 1.000 | 0.916 | 0.941 | n/s | 0.540 | 0.645 | 0.750 | < 0.0001 |
|  |  | e=10 | 0.916 | 0.911 | 0.931 | n/s | 0.536 | 0.634 | 0.736 | < 0.0001 |
|  |  | e=6 | 1.33 | 1.72 | 1.61 | 0.00237 | 0.90 | 1.17 | 1.31 | < 0.0001 |
|  | 6+ | perfect | 0 | 0.0121 | 0.00740 | n/s | 0.00772 | 0.00293 | 0.00262 | n/s |
|  |  | e=10 | 0.0572 | 0.00605 | 0.0171 | n/s | 0.00772 | 0.00837 | 0.0118 | n/s |
|  |  | e=6 | 0.0572 | 0.0371 | 0.0171 | n/s | 0.0282 | 0.0219 | 0.0361 | 0.00402 |
|  | 10+ | perfect | 0 | 0 | 0 | n/a | 0 | 0.00133 | 0 | n/s |
|  |  | e=10 | 0 | 0.006047 | 0 | n/s | 0 | 0.00221 | 0 | n/s |
|  |  | e=6 | 0 | 0.006047 | 0 | n/s | 0 | 0.00221 | 0.00046 | n/s |
| 2 (CG) | 2 | perfect | 1.38 | 1.78 | 1.98 | 0.00155 | 0.89 | 1.59 | 1.70 | < 0.0001 |
|  |  | e=10 | 1.38 | 1.78 | 1.98 | 0.00155 | 0.89 | 1.59 | 1.70 | < 0.0001 |
|  |  | e=6 | 1.19 | 1.68 | 1.90 | 0.00032 | 0.87 | 1.54 | 1.63 | < 0.0001 |
|  | 3 to 5 | perfect | 0.00507 | 0.16 | 0.18 | n/s | 0.0542 | 0.0938 | 0.0864 | < 0.0001 |
|  |  | e=10 | 0.00507 | 0.16 | 0.18 | n/s | 0.0542 | 0.0937 | 0.0864 | < 0.0001 |
|  |  | e=6 | 0.0894 | 0.23 | 0.26 | n/s | 0.0719 | 0.138 | 0.145 | < 0.0001 |
|  | 6+ | perfect | 0 | 0 | 0 | n/a | 0 | 0 | 0 | n/a |
|  |  | e=10 | 0 | 0 | 0 | n/a | 0 | 0.00011 | 0 | n/s |
|  |  | e=6 | 0 | 0 | 0 | n/a | 0 | 0.00011 | 0 | n/s |
|  | 10+ | perfect | 0 | 0 | 0 | n/a | 0 | 0 | 0 | n/a |
|  |  | e=10 | 0 | 0 | 0 | n/a | 0 | 0 | 0 | n/a |
|  |  | e=6 | 0 | 0 | 0 | n/a | 0 | 0 | 0 | n/a |
| 2 (all  motifs) | 2 | perfect | 24.1 | 23.5 | 24.2 | n/s | 24.5 | 24.3 | 24.3 | n/s |
|  |  | e=10 | 23.6 | 23.3 | 24.0 | n/s | 24.2 | 24.1 | 24.2 | n/s |
|  |  | e=6 | 20.0 | 20.6 | 21.6 | n/s | 20.9 | 21.4 | 21.7 | 0.000311 |
|  | 3 to 5 | perfect | 6.27 | 4.48 | 3.87 | n/s | 5.38 | 3.72 | 2.92 | < 0.0001 |
|  |  | e=10 | 5.76 | 4.18 | 3.63 | n/s | 4.91 | 3.51 | 2.76 | < 0.0001 |
|  |  | e=6 | 7.47 | 6.01 | 5.56 | n/s | 6.85 | 5.34 | 4.50 | < 0.0001 |
|  | 6+ | perfect | 0.957 | 0.211 | 0.230 | n/s | 0.403 | 0.153 | 0.0597 | < 0.0001 |
|  |  | e=10 | 1.38 | 0.393 | 0.413 | n/s | 0.675 | 0.281 | 0.169 | < 0.0001 |
|  |  | e=6 | 1.84 | 0.616 | 0.385 | n/s | 1.03 | 0.413 | 0.221 | < 0.0001 |
|  | 10+ | perfect | 0.622 | 0.0378 | 0.0450 | n/s | 0.141 | 0.0444 | 0.00908 | 0.00019 |
|  |  | e=10 | 0.754 | 0.0816 | 0.0935 | n/s | 0.214 | 0.0653 | 0.0232 | < 0.0001 |
|  |  | e=6 | 0.760 | 0.118 | 0.131 | n/s | 0.226 | 0.0801 | 0.0283 | 0.00227 |
| 3 (all  motifs) | 2 | perfect | 9.45 | 10.9 | 12.0 | n/s | 11.2 | 11.2 | 11.5 | n/s |
|  |  | e=10 | 9.20 | 10.7 | 11.9 | 0.00491 | 11.0 | 11.1 | 11.4 | 0.002616 |
|  |  | e=6 | 7.23 | 9.25 | 10.8 | < 0.0001 | 9.40 | 9.73 | 10.1 | < 0.0001 |
|  | 3 to 5 | perfect | 1.000 | 0.559 | 0.614 | n/s | 0.643 | 0.539 | 0.446 | 0.00108 |
|  |  | e=10 | 0.871 | 0.527 | 0.569 | n/s | 0.586 | 0.525 | 0.438 | 0.00025 |
|  |  | e=6 | 2.58 | 1.96 | 1.57 | n/s | 2.10 | 1.84 | 1.78 | n/s |

**Table S13:** continued

| **Repeat type** | | | **Mean per kb repeat**  **frequencies for hot IGRs** | | | | **Mean per kb repeat**  **frequencies for non hot IGRs** | | | |
| --- | --- | --- | --- | --- | --- | --- | --- | --- | --- | --- |
| Motif  length | Copy  number | Mismatch  type | Number of promoters | | | P value | Number of promoters | | | P value |
|  |  |  | None | One | Two |  | None | One | Two |  |
| 3 (all  motifs) | 6+ | perfect | 0.0875 | 0.0149 | 0.0714 | n/s | 0.0269 | 0.00821 | 0.00573 | n/s |
|  |  | e=10 | 0.111 | 0.0440 | 0.0619 | n/s | 0.0644 | 0.0175 | 0.0127 | n/s |
|  |  | e=6 | 0.138 | 0.102 | 0.100 | n/s | 0.109 | 0.0361 | 0.0258 | n/s |
|  | 10+ | perfect | 0.0478 | 0 | 0 | n/s | 0.0159 | 0.00215 | 0.00130 | n/s |
|  |  | e=10 | 0.0875 | 0.00744 | 0 | n/s | 0.0265 | 0.00641 | 0.00130 | n/s |
|  |  | e=6 | 0.0875 | 0.00744 | 0 | n/s | 0.0275 | 0.00811 | 0.00130 | n/s |
| 4 (all  motifs) | 2 | perfect | 5.36 | 4.33 | 3.72 | n/s | 4.45 | 3.93 | 3.56 | n/s |
|  |  | e=10 | 5.14 | 4.13 | 3.58 | n/s | 4.13 | 3.75 | 3.43 | n/s |
|  |  | e=6 | 4.04 | 3.47 | 2.98 | n/s | 3.14 | 3.10 | 2.86 | 0.00017 |
|  | 3 to 5 | perfect | 0.0653 | 0.209 | 0.111 | n/s | 0.150 | 0.105 | 0.0664 | n/s |
|  |  | e=10 | 0.119 | 0.374 | 0.207 | n/s | 0.454 | 0.245 | 0.190 | n/s |
|  |  | e=6 | 0.233 | 0.419 | 0.240 | n/s | 0.568 | 0.319 | 0.258 | n/s |
|  | 6+ | perfect | 0 | 0 | 0 | n/a | 0.0152 | 0.00167 | 0.00096 | n/s |
|  |  | e=10 | 0 | 0 | 0 | n/a | 0.0116 | 0.00523 | 0.00096 | n/s |
|  |  | e=6 | 0 | 0 | 0 | n/a | 0.0155 | 0.00825 | 0.00199 | n/s |
|  | 10+ | perfect | 0 | 0 | 0 | n/a | 0.00593 | 0 | 0 | n/s |
|  |  | e=10 | 0 | 0 | 0 | n/a | 0.00593 | 0 | 0 | n/s |
|  |  | e=6 | 0 | 0 | 0 | n/a | 0.00593 | 0 | 0 | n/s |
| 5 (all  motifs) | 2 | perfect | 2.03 | 1.61 | 1.72 | n/s | 1.75 | 1.52 | 1.45 | 0.00066 |
|  |  | e=10 | 2.05 | 1.51 | 1.56 | n/s | 1.57 | 1.41 | 1.33 | < 0.0001 |
|  |  | e=6 | 1.49 | 1.18 | 1.28 | n/s | 1.18 | 1.13 | 1.02 | < 0.0001 |
|  | 3 to 5 | perfect | 0.0370 | 0.0734 | 0.0119 | n/s | 0.0326 | 0.0310 | 0.0351 | n/s |
|  |  | e=10 | 0.118 | 0.126 | 0.0411 | n/s | 0.118 | 0.0899 | 0.0956 | n/s |
|  |  | e=6 | 0.0809 | 0.117 | 0.0378 | n/s | 0.106 | 0.0957 | 0.0981 | n/s |
|  | 6+ | perfect | 0 | 0 | 0 | n/a | 0 | 0.00109 | 0.00112 | n/s |
|  |  | e=10 | 0 | 0 | 0.00474 | n/s | 0 | 0.00109 | 0.00112 | n/s |
|  |  | e=6 | 0 | 0 | 0.00474 | n/s | 0.00217 | 0.00152 | 0.00527 | n/s |
|  | 10+ | perfect | 0 | 0 | 0 | n/a | 0 | 0 | 0 | n/a |
|  |  | e=10 | 0 | 0 | 0 | n/a | 0 | 0 | 0 | n/a |
|  |  | e=6 | 0 | 0 | 0 | n/a | 0 | 0 | 0 | n/a |
| 6 (all  motifs) | 2 | perfect | 1.03 | 0.705 | 0.841 | n/s | 0.825 | 0.606 | 0.495 | n/s |
|  |  | e=10 | 0.861 | 0.650 | 0.768 | n/s | 0.724 | 0.550 | 0.457 | n/s |
|  |  | e=6 | 0.385 | 0.507 | 0.630 | n/s | 0.484 | 0.375 | 0.327 | n/s |
|  | 3 to 5 | perfect | 0.0557 | 0.0655 | 0.0157 | n/s | 0.0223 | 0.0160 | 0.0200 | n/s |
|  |  | e=10 | 0.0309 | 0.0538 | 0.0596 | n/s | 0.0366 | 0.0454 | 0.0298 | n/s |
|  |  | e=6 | 0.00310 | 0.0507 | 0.0503 | n/s | 0.0344 | 0.0306 | 0.0209 | n/s |
|  | 6+ | perfect | 0 | 0.0109 | 0 | n/s | 0 | 0.00087 | 0 | n/s |
|  |  | e=10 | 0 | 0.0109 | 0 | n/s | 0 | 0.00087 | 0.00191 | n/s |
|  |  | e=6 | 0 | 0.0109 | 0 | n/s | 0.00207 | 0.00324 | 0.00240 | n/s |
|  | 10+ | perfect | 0 | 0 | 0 | n/a | 0 | 0 | 0 | n/a |
|  |  | e=10 | 0 | 0 | 0 | n/a | 0 | 0 | 0.000662 | n/s |
|  |  | e=6 | 0 | 0 | 0 | n/a | 0 | 0 | 0.000662 | n/s |

### Table S14: Numbers of microsatellites, in IGRs, found within five or ten bp of other microsatellites of the same or larger size group (compound and degenerate repeats), including the subset of these which had repeat motifs with the same base composition (degenerate repeats). IGRs were divided by recombination (double-strand break) intensity as reported by Gerton and co-workers [1] into 473 hot and and 5520 non-hot regions. Imperfect repeats were allowed, with a maximum of one mismatch per six bp. Degenerate repeats only were considered for microsatellites with less than six copies. This was because results would be affected to an inordinate degree by the fact that short poly-A arrays are extremely abundant relative to other repeat types.

| **Repeat type** | | **Total number**  **of repeats** | | Compound & degenerate repeats | | | | Degenerate repeats only | | | |
| --- | --- | --- | --- | --- | --- | --- | --- | --- | --- | --- | --- |
|  |  |  |  | % within 5 bp of  another repeat | | % within 10 bp of  another repeat | | % within 5 bp of  another repeat | | % within 10 bp of  another repeat | |
| Motif  length | Copy  number |  |  |  |  |  |  |  |  |  |  |
|  |  | Hot | Non-hot | Hot | Non hot | Hot | Non hot | Hot | Non-hot | Hot | Non-hot |
| 1 (A) | 3 to 5 | 8459 | 106082 | n/a | n/a | n/a | n/a | 39.1 | 41.2 | 61.5 | 64.3 |
|  | 6+ | 1473 | 15164 | 12.4 | 10.1 | 20.1 | 17.0 | 11.7 | 9.6 | 18.6 | 15.9 |
|  | 14+ | 173 | 919 | 16.2 | 15.0 | 23.7 | 24.3 | 14.5 | 14.5 | 20.8 | 22.9 |
| 1 (G) | 3 to 5 | 2428 | 23094 | n/a | n/a | n/a | n/a | 13.2 | 9.44 | 24.1 | 19.3 |
|  | 6+ | 46 | 305 | 8.70 | 6.89 | 13.0 | 11.8 | 0 | 0 | 0 | 0.656 |
|  | 14+ | 2 | 2 | 0 | 0 | 0 | 0 | 0 | 0 | 0 | 0 |
| 2 (all  motifs) | 2 | 5586 | 63536 | n/a | n/a | n/a | n/a | 8.65 | 9.11 | 17.7 | 17.0 |
|  | 3 to 5 | 1412 | 14380 | n/a | n/a | n/a | n/a | 4.67 | 4.44 | 7.08 | 7.48 |
|  | 6+ | 130 | 1022 | 8.46 | 11.1 | 12.3 | 18.8 | 0.769 | 3.33 | 0.769 | 4.40 |
|  | 10+ | 33 | 209 | 3.03 | 13.4 | 12.1 | 19.1 | 0 | 5.74 | 0 | 6.70 |
| 3 (all  motifs) | 2 | 2682 | 29846 | n/a | n/a | n/a | n/a | 2.16 | 1.88 | 3.91 | 3.81 |
|  | 3 to 5 | 520 | 5524 | n/a | n/a | n/a | n/a | 0.385 | 0.597 | 1.15 | 1.30 |
|  | 6+ | 21 | 118 | 9.52 | 15.3 | 19.0 | 22.0 | 0 | 5.08 | 0 | 5.08 |
|  | 10+ | 3 | 20 | 0 | 10 | 0 | 25 | 0 | 5 | 0 | 5 |
| 4 (all  motifs) | 2 | 810 | 8568 | n/a | n/a | n/a | n/a | 0.123 | 0.397 | 1.60 | 0.794 |
|  | 3 to 5 | 76 | 828 | n/a | n/a | n/a | n/a | 0 | 0.242 | 0 | 1.45 |
|  | 6+ | 0 | 19 | n/a | 0 | n/a | 0 | n/a | 0 | n/a | 0 |
|  | 10+ | 0 | 1 | n/a | 0 | n/a | 0 | n/a | 0 | n/a | 0 |
| 5 (all  motifs) | 2 | 298 | 3175 | n/a | n/a | n/a | n/a | 0 | 0.126 | 0 | 0.126 |
|  | 3 to 5 | 26 | 272 | n/a | n/a | n/a | n/a | 0 | 0 | 0 | 0 |
|  | 6+ | 1 | 5 | 0 | 0 | 0 | 0 | 0 | 0 | 0 | 0 |
|  | 10+ | 0 | 0 | n/a | n/a | n/a | n/a | n/a | n/a | n/a | n/a |
| 6 (all  motifs) | 2 | 123 | 1074 | n/a | n/a | n/a | n/a | 1.63 | 0.372 | 1.63 | 0.372 |
|  | 3 to 5 | 8 | 69 | n/a | n/a | n/a | n/a | 0 | 0 | 0 | 0 |
|  | 6+ | 1 | 10 | 0 | 20 | 0 | 20 | 0 | 0 | 0 | 0 |
|  | 10+ | 0 | 4 | n/a | 25 | n/a | 25 | n/a | 0 | n/a | 0 |

**Table S15:** Numbers of microsatellites, in ORFs, found within five or ten bp of other microsatellites of the same or larger size group (compound and degenerate repeats), including the subset of these which had repeat motifs with the same base composition (degenerate repeats). ORFs were divided by recombination (double-strand break) intensity as reported by Gerton and co-workers [1] into 297 hot and 5683 non-hot regions. Imperfect repeats were allowed, wih a maximum of one mismatch per six bp. Degenerate repeats only were considered for microsatellites with less than six copies. This was because results would be affected to an inordinate degree by the fact that short poly-A arrays are extremely abundant relative to other repeat types.

| **Repeat type** | | **Total number**  **of repeats** | | **Compound & degenerate repeats** | | | | **Degenerate repeats only** | | | |
| --- | --- | --- | --- | --- | --- | --- | --- | --- | --- | --- | --- |
|  |  |  |  | % within 5 bp of  another repeat | | % within 10 bp of  another repeat | | % within 5 bp of  another repeat | | % within 10 bp of  another repeat | |
| Motif  length | Copy  number |  |  |  |  |  |  |  |  |  |  |
|  |  | Hot | Non-hot | Hot | Non-hot | Hot | Non-hot | Hot | Non-hot | Hot | Non-hot |
| 1 (A) | 3 to 5 | 9926 | 303316 | n/a | n/a | n/a | n/a | 31.3 | 36.9 | 51.5 | 59.7 |
|  | 6+ | 436 | 17655 | 2.06 | 3.37 | 4.82 | 6.31 | 1.83 | 3.21 | 4.36 | 5.99 |
|  | 14+ | 7 | 131 | 28.6 | 9.16 | 42.9 | 14.5 | 14.3 | 8.40 | 28.6 | 13.0 |
| 1 (G) | 3 to 5 | 4419 | 80999 | n/a | n/a | n/a | n/a | 11.5 | 8.88 | 24.7 | 18.0 |
|  | 6+ | 44 | 642 | 2.27 | 2.49 | 2.27 | 4.83 | 0 | 0 | 0 | 0 |
|  | 14+ | 0 | 0 | n/a | n/a | n/a | n/a | n/a | n/a | n/a | n/a |
| 2 (all  motifs) | 2 | 7548 | 178401 | n/a | n/a | n/a | n/a | 7.94 | 7.16 | 15.7 | 14.5 |
|  | 3 to 5 | 1047 | 25924 | n/a | n/a | n/a | n/a | 1.81 | 1.18 | 2.77 | 2.37 |
|  | 6+ | 24 | 245 | 12.5 | 6.53 | 20.8 | 11.0 | 0 | 0.408 | 8.33 | 0.408 |
|  | 10+ | 8 | 19 | 25 | 10.5 | 37.5 | 21.1 | 0 | 0 | 12.5 | 0 |
| 3 (all  motifs) | 2 | 4302 | 105759 | n/a | n/a | n/a | n/a | 1.88 | 1.71 | 3.25 | 3.64 |
|  | 3 to 5 | 677 | 15459 | n/a | n/a | n/a | n/a | 1.48 | 1.05 | 2.36 | 1.95 |
|  | 6+ | 28 | 478 | 3.57 | 9.62 | 3.57 | 14.4 | 0 | 3.97 | 0 | 3.97 |
|  | 10+ | 0 | 100 | n/a | 16 | n/a | 20 | n/a | 10 | n/a | 10 |
| 4 (all  motifs) | 2 | 741 | 20103 | n/a | n/a | n/a | n/a | 0 | 0.139 | 0 | 0.269 |
|  | 3 to 5 | 38 | 856 | n/a | n/a | n/a | n/a | 0 | 0 | 0 | 0.234 |
|  | 6+ | 0 | 2 | n/a | 0 | n/a | 0 | n/a | 0 | n/a | 0 |
|  | 10+ | 0 | 0 | n/a | n/a | n/a | n/a | n/a | n/a | n/a | n/a |
| 5 (all  motifs) | 2 | 215 | 6024 | n/a | n/a | n/a | n/a | 0 | 0.0498 | 0 | 0.116 |
|  | 3 to 5 | 1 | 137 | n/a | n/a | n/a | n/a | 0 | 0 | 0 | 0 |
|  | 6+ | 0 | 4 | n/a | 25 | n/a | 25 | n/a | 0 | n/a | 0 |
|  | 10+ | 0 | 1 | n/a | 100 | n/a | 100 | n/a | 0 | n/a | 0 |
| 6 (all  motifs) | 2 | 159 | 3389 | n/a | n/a | n/a | n/a | 0 | 0.177 | 1.26 | 0.354 |
|  | 3 to 5 | 18 | 149 | n/a | n/a | n/a | n/a | 0 | 0 | 0 | 0.671 |
|  | 6+ | 4 | 11 | 0 | 9.09 | 0 | 27.3 | 0 | 0 | 0 | 18.2 |
|  | 10+ | 1 | 5 | 0 | 20 | 0 | 60 | 0 | 0 | 0 | 40 |

**Table S16:** Mean microsatellite frequencies in hot IGRs and flanking IGRs one and two ORFs removed from hotspots. Statistical comparisons were made between the flanking IGRs and non-hot IGRs more than four ORFs removed from hotspots**.** All p values <0.01 are shown, but caution is recommended in view of the multiple hypotheses being tested.

| **Repeat type** | | | **Mean repeat frequency by IGR type** | | | | **P value** | |
| --- | --- | --- | --- | --- | --- | --- | --- | --- |
| Motif  length | Copy  number | Mismatch  type | Hot | 1 removed  from hot | 2 removed  from hot | Non hot | 1 removed  v non hot | 2 removed  v non hot |
| 1 (A) | 3 to 5 | perfect | 35 | 40.4 | 40.7 | 39.8 | n/s | n/s |
|  |  | e=10 | 34.3 | 39.8 | 40 | 39.4 | n/s | n/s |
|  |  | e=6 | 31.8 | 36.8 | 37.4 | 36.7 | n/s | n/s |
|  | 6+ | perfect | 5.42 | 4.93 | 5.28 | 4.51 | n/s | 0.0037 |
|  |  | e=10 | 5.24 | 4.79 | 5.19 | 4.4 | n/s | 0.0025 |
|  |  | e=6 | 6.12 | 6.06 | 6.21 | 5.42 | n/s | 0.00432 |
|  | 14+ | perfect | 0.418 | 0.288 | 0.21 | 0.165 | n/s | n/s |
|  |  | e=10 | 0.733 | 0.631 | 0.372 | 0.292 | 0.00027 | n/s |
|  |  | e=6 | 0.854 | 0.773 | 0.412 | 0.353 | 0.00029 | n/s |
| 1 (G) | 3 to 5 | perfect | 9.18 | 7.38 | 6.13 | 7.32 | n/s | 0.00022 |
|  |  | e=10 | 9.16 | 7.35 | 6.12 | 7.31 | n/s | 0.00024 |
|  |  | e=6 | 8.89 | 7.22 | 6.14 | 7.18 | n/s | 0.00094 |
|  | 6+ | perfect | 0.118 | 0.0802 | 0.0672 | 0.0739 | n/s | n/s |
|  |  | e=10 | 0.114 | 0.0802 | 0.0672 | 0.0723 | n/s | n/s |
|  |  | e=6 | 0.16 | 0.092 | 0.0806 | 0.0914 | n/s | n/s |
|  | 14+ | perfect | 0.0035 | 0 | 0 | 0.00093 | n/s | n/s |
|  |  | e=10 | 0.0035 | 0 | 0 | 0.00093 | n/s | n/s |
|  |  | e=6 | 0.0035 | 0 | 0 | 0.00093 | n/s | n/s |
| 2 (AT) | 2 | perfect | 7.69 | 8.93 | 9.61 | 9.22 | n/s | n/s |
|  |  | e=10 | 7.57 | 8.77 | 9.42 | 9.1 | n/s | n/s |
|  |  | e=6 | 6.26 | 6.97 | 7.8 | 7.63 | n/s | n/s |
|  | 3 to 5 | perfect | 2.68 | 2.82 | 2.76 | 2.59 | n/s | n/s |
|  |  | e=10 | 2.42 | 2.61 | 2.61 | 2.43 | n/s | n/s |
|  |  | e=6 | 3.03 | 3.61 | 3.55 | 3.21 | n/s | n/s |
|  | 6+ | perfect | 0.308 | 0.331 | 0.148 | 0.156 | n/s | n/s |
|  |  | e=10 | 0.45 | 0.346 | 0.207 | 0.224 | n/s | n/s |
|  |  | e=6 | 0.627 | 0.611 | 0.485 | 0.435 | n/s | n/s |
|  | 10+ | perfect | 0.142 | 0.046 | 0.0547 | 0.0505 | n/s | n/s |
|  |  | e=10 | 0.197 | 0.205 | 0.0848 | 0.0732 | n/s | n/s |
|  |  | e=6 | 0.221 | 0.237 | 0.0948 | 0.082 | n/s | n/s |
| 2 (AC) | 2 | perfect | 6.8 | 6.37 | 6.67 | 6.61 | n/s | n/s |
|  |  | e=10 | 6.69 | 6.35 | 6.65 | 6.56 | n/s | n/s |
|  |  | e=6 | 6.13 | 5.83 | 6.21 | 6.01 | n/s | n/s |
|  | 3 to 5 | perfect | 0.908 | 0.709 | 0.425 | 0.595 | n/s | 0.0099 |
|  |  | e=10 | 0.924 | 0.689 | 0.425 | 0.578 | n/s | n/s |
|  |  | e=6 | 1.32 | 1.08 | 0.774 | 0.992 | n/s | n/s |
|  | 6+ | perfect | 0.0518 | 0.0064 | 0.0167 | 0.0167 | n/s | n/s |
|  |  | e=10 | 0.0772 | 0.0064 | 0.0167 | 0.0223 | n/s | n/s |
|  |  | e=6 | 0.134 | 0.0292 | 0.0602 | 0.0469 | n/s | n/s |
|  | 10+ | perfect | 0.0159 | 0 | 0.0114 | 0.00291 | n/s | n/s |
|  |  | e=10 | 0.0218 | 0 | 0.0114 | 0.00336 | n/s | n/s |
|  |  | e=6 | 0.0283 | 0 | 0.00362 | 0.00876 | n/s | n/s |

Table S16: continued

| **Repeat type** | | | **Mean repeat frequency by IGR type** | | | | **P value** | |
| --- | --- | --- | --- | --- | --- | --- | --- | --- |
| Motif  length | Copy  number | Mismatch  type | Hot | 1 removed  from hot | 2removed  from hot | Non hot | 1 removed  v non hot | 2 removed  v non hot |
| 2 (AG) | 2 | perfect | 7.57 | 6.47 | 6.52 | 7.19 | 0.00752 | n/s |
|  |  | e=10 | 7.53 | 6.44 | 6.53 | 7.17 | 0.00688 | n/s |
|  |  | e=6 | 6.73 | 5.82 | 5.78 | 6.47 | n/s | n/s |
|  | 3 to 5 | perfect | 0.94 | 0.687 | 0.681 | 0.656 | n/s | n/s |
|  |  | e=10 | 0.918 | 0.655 | 0.679 | 0.647 | n/s | n/s |
|  |  | e=6 | 1.61 | 1.12 | 1.2 | 1.17 | n/s | n/s |
|  | 6+ | perfect | 0.00828 | 0.0164 | 0.0152 | 0.00117 | 0.00027 | n/s |
|  |  | e=10 | 0.0196 | 0.0322 | 0.0152 | 0.00705 | n/s | n/s |
|  |  | e=6 | 0.03541 | 0.08742 | 0.0222 | 0.0241 | n/s | n/s |
|  | 10+ | perfect | 0 | 0.00954 | 0 | < 0.0001 | < 0.0001 | n/s |
|  |  | e=10 | 0.00307 | 0.0164 | 0 | < 0.0001 | < 0.0001 | n/s |
|  |  | e=6 | 0.00307 | 0.0164 | 0 | 0.00022 | < 0.0001 | n/s |
| 2 (CG) | 2 | perfect | 1.76 | 1.25 | 1.4 | 1.45 | 0.00978 | n/s |
|  |  | e=10 | 1.76 | 1.24 | 1.4 | 1.45 | 0.00761 | n/s |
|  |  | e=6 | 1.64 | 1.17 | 1.3 | 1.4 | 0.00375 | n/s |
|  | 3 to 5 | perfect | 0.132 | 0.0979 | 0.0412 | 0.0886 | n/s | n/s |
|  |  | e=10 | 0.132 | 0.0979 | 0.0412 | 0.0886 | n/s | n/s |
|  |  | e=6 | 0.213 | 0.148 | 0.0766 | 0.131 | n/s | n/s |
|  | 6+ | perfect | 0 | 0 | 0 | 0 | n/a | n/a |
|  |  | e=10 | 0 | 0 | 0 | < 0.0001 | n/s | n/s |
|  |  | e=6 | 0 | 0 | 0 | < 0.0001 | n/s | n/s |
|  | 10+ | perfect | 0 | 0 | 0 | 0 | n/a | n/a |
|  |  | e=10 | 0 | 0 | 0 | 0 | n/a | n/a |
|  |  | e=6 | 0 | 0 | 0 | 0 | n/a | n/a |
| 2 (all  motifs) | 2 | perfect | 23.8 | 23 | 24.2 | 24.5 | 0.00654 | n/s |
|  |  | e=10 | 23.5 | 22.8 | 24 | 24.3 | 0.00482 | n/s |
|  |  | e=6 | 20.8 | 19.8 | 21.1 | 21.5 | 0.00081 | n/s |
|  | 3 to 5 | perfect | 4.67 | 4.31 | 3.91 | 3.93 | n/s | n/s |
|  |  | e=10 | 4.34 | 3.93 | 3.63 | 3.67 | n/s | n/s |
|  |  | e=6 | 6.17 | 5.95 | 5.6 | 5.5 | n/s | n/s |
|  | 6+ | perfect | 0.368 | 0.354 | 0.18 | 0.174 | n/s | n/s |
|  |  | e=10 | 0.599 | 0.514 | 0.362 | 0.332 | n/s | n/s |
|  |  | e=6 | 0.797 | 0.727 | 0.567 | 0.506 | n/s | n/s |
|  | 10+ | perfect | 0.158 | 0.0555 | 0.0661 | 0.0535 | n/s | n/s |
|  |  | e=10 | 0.221 | 0.222 | 0.0963 | 0.0766 | n/s | n/s |
|  |  | e=6 | 0.252 | 0.253 | 0.0984 | 0.091 | n/s | n/s |
| 3 (all  motifs) | 2 | perfect | 10.9 | 11.2 | 11.4 | 11.2 | n/s | n/s |
|  |  | e=10 | 10.8 | 11 | 11.2 | 11.1 | n/s | n/s |
|  |  | e=6 | 9.29 | 9.32 | 9.76 | 9.7 | n/s | n/s |
|  | 3 to 5 | perfect | 0.664 | 0.448 | 0.492 | 0.541 | n/s | n/s |
|  |  | e=10 | 0.609 | 0.437 | 0.392 | 0.525 | n/s | n/s |
|  |  | e=6 | 1.97 | 1.83 | 1.68 | 1.91 | n/s | n/s |
|  | 6+ | perfect | 0.046 | 0.0416 | 0.0156 | 0.00875 | n/s | n/s |
|  |  | e=10 | 0.0627 | 0.0416 | 0.0495 | 0.0219 | n/s | n/s |
|  |  | e=6 | 0.109 | 0.123 | 0.0997 | 0.04 | n/s | n/s |

Table S16: continued

| **Repeat type** | | | **Mean repeat frequency by IGR type** | | | | **P value** | |
| --- | --- | --- | --- | --- | --- | --- | --- | --- |
| Motif  length | Copy  number | Mismatch  type | Hot | 1 removed  from hot | 2 removed  from hot | Non hot | 1 removed  v non hot | 2 removed  v non hot |
| 3 (all  motifs) | 10+ | perfect | 0.0097 | 0.0378 | 0.00881 | 0.00344 | n/s | n/s |
|  |  | e=10 | 0.0215 | 0.0416 | 0.017 | 0.00781 | n/s | n/s |
|  |  | e=6 | 0.0215 | 0.0416 | 0.0265 | 0.00816 | n/s | n/s |
| 4 (all  motifs) | 2 | perfect | 4.36 | 4.14 | 3.81 | 3.97 | n/s | n/s |
|  |  | e=10 | 4.17 | 4 | 3.54 | 3.77 | n/s | n/s |
|  |  | e=6 | 3.44 | 3.21 | 2.77 | 3.06 | n/s | n/s |
|  | 3 to 5 | perfect | 0.151 | 0.0986 | 0.18 | 0.098 | n/s | n/s |
|  |  | e=10 | 0.274 | 0.297 | 0.431 | 0.267 | n/s | n/s |
|  |  | e=6 | 0.329 | 0.376 | 0.507 | 0.35 | n/s | n/s |
|  | 6+ | perfect | 0 | 0.0254 | 0 | 0.00443 | n/s | n/s |
|  |  | e=10 | 0 | 0.0276 | 0.00834 | 0.00276 | n/s | n/s |
|  |  | e=6 | 0 | 0.0276 | 0.0106 | 0.00461 | n/s | n/s |
|  | 10+ | perfect | 0 | 0.0254 | 0 | 0 | 0.00035 | n/a |
|  |  | e=10 | 0 | 0.0254 | 0 | 0 | 0.00035 | n/a |
|  |  | e=6 | 0 | 0.0254 | 0 | 0 | 0.00035 | n/a |
| 5 (all  motifs) | 2 | perfect | 1.72 | 1.65 | 1.73 | 1.56 | n/s | n/s |
|  |  | e=10 | 1.63 | 1.48 | 1.56 | 1.43 | n/s | n/s |
|  |  | e=6 | 1.28 | 1.08 | 1.22 | 1.1 | n/s | n/s |
|  | 3 to 5 | perfect | 0.0482 | 0.0418 | 0.0354 | 0.0357 | n/s | n/s |
|  |  | e=10 | 0.0999 | 0.108 | 0.144 | 0.1 | n/s | n/s |
|  |  | e=6 | 0.0867 | 0.115 | 0.126 | 0.0959 | n/s | n/s |
|  | 6+ | perfect | 0 | 0 | 0 | 0.00103 | n/s | n/s |
|  |  | e=10 | 0.00137 | 0 | 0 | 0.00134 | n/s | n/s |
|  |  | e=6 | 0.00137 | 0.0205 | 0 | 0.00176 | 0.0047 | n/s |
|  | 10+ | perfect | 0 | 0 | 0 | 0 | n/a | n/a |
|  |  | e=10 | 0 | 0 | 0 | 0 | n/a | n/a |
|  |  | e=6 | 0 | 0 | 0 | 0 | n/a | n/a |
| 6 (all  motifs) | 2 | perfect | 0.811 | 0.834 | 0.721 | 0.64 | n/s | n/s |
|  |  | e=10 | 0.727 | 0.718 | 0.647 | 0.563 | n/s | n/s |
|  |  | e=6 | 0.518 | 0.523 | 0.424 | 0.387 | n/s | n/s |
|  | 3 to 5 | perfect | 0.0491 | 0.0534 | 0.0313 | 0.0203 | n/s | n/s |
|  |  | e=10 | 0.0509 | 0.0916 | 0.0407 | 0.0429 | n/s | n/s |
|  |  | e=6 | 0.0409 | 0.025 | 0.0485 | 0.0297 | n/s | n/s |
|  | 6+ | perfect | 0.00552 | 0 | 0 | 0.00079 | n/s | n/s |
|  |  | e=10 | 0.00552 | 0 | 0 | 0.00578 | n/s | n/s |
|  |  | e=6 | 0.00552 | 0 | 0 | 0.00291 | n/s | n/s |
|  | 10+ | perfect | 0 | 0 | 0 | 0 | n/a | n/a |
|  |  | e=10 | 0 | 0 | 0 | 0.00228 | n/s | n/s |
|  |  | e=6 | 0 | 0 | 0 | 0.00112 | n/s | n/s |

**Table S17:** Mean microsatellite frequencies in hot ORFs and flanking ORFs one and two IGRs removed from hotspots. Statistical comparisons were made between the flanking ORFs and non-hot ORFs more than four IGRs removed from hotspots**.** All p values <0.01 are shown, but caution is recommended in view of the multiple hypotheses being tested.

| **Repeat type** | | | **Mean repeat frequency by ORF type** | | | | **P value** | |
| --- | --- | --- | --- | --- | --- | --- | --- | --- |
| Motif  length | Copy  number | Mismatch  type | Hot | 1 removed  from hot | 2 removed  from hot | Non-hot | 1 removed  v non-hot | 2 removed  v non-hot |
| 1 (A) | 3 to 5 | perfect | 29.2 | 34 | 36.6 | 36.1 | < 0.0001 | n/s |
|  |  | e=10 | 29.1 | 33.9 | 36.5 | 36 | < 0.0001 | n/s |
|  |  | e=6 | 28 | 32.7 | 34.9 | 34.7 | < 0.0001 | n/s |
|  | 6+ | perfect | 0.981 | 1.38 | 1.59 | 1.66 | 0.0006 | n/s |
|  |  | e=10 | 0.978 | 1.38 | 1.59 | 1.65 | 0.0007 | n/s |
|  |  | e=6 | 1.28 | 1.79 | 2.2 | 2.15 | 0.00013 | n/s |
|  | 14+ | perfect | 0.0134 | 0.00657 | 0.0329 | 0.00589 | n/s | n/s |
|  |  | e=10 | 0.0182 | 0.0211 | 0.0488 | 0.0148 | n/s | n/s |
|  |  | e=6 | 0.0218 | 0.0251 | 0.0603 | 0.0266 | n/s | n/s |
| 1 (G) | 3 to 5 | perfect | 12.9 | 11.1 | 9.93 | 10 | < 0.0001 | n/s |
|  |  | e=10 | 12.9 | 11.1 | 9.91 | 10 | < 0.0001 | n/s |
|  |  | e=6 | 12.6 | 10.9 | 9.8 | 9.87 | < 0.0001 | n/s |
|  | 6+ | perfect | 0.0992 | 0.0878 | 0.0388 | 0.0657 | n/s | n/s |
|  |  | e=10 | 0.0992 | 0.0878 | 0.0388 | 0.0657 | n/s | n/s |
|  |  | e=6 | 0.135 | 0.107 | 0.0476 | 0.0826 | n/s | n/s |
|  | 14+ | perfect | 0 | 0 | 0 | 0 | n/a | n/a |
|  |  | e=10 | 0 | 0 | 0 | 0 | n/a | n/a |
|  |  | e=6 | 0 | 0 | 0 | 0 | n/a | n/a |
| 2 (AT) | 2 | perfect | 4.46 | 5.64 | 5.91 | 5.93 | n/s | n/s |
|  |  | e=10 | 4.43 | 5.63 | 5.89 | 5.91 | n/s | n/s |
|  |  | e=6 | 4.07 | 5.19 | 5.35 | 5.39 | n/s | n/s |
|  | 3 to 5 | perfect | 0.434 | 0.488 | 0.586 | 0.592 | n/s | n/s |
|  |  | e=10 | 0.409 | 0.486 | 0.586 | 0.587 | n/s | n/s |
|  |  | e=6 | 0.655 | 0.767 | 0.979 | 0.979 | 0.0044 | n/s |
|  | 6+ | perfect | 0.0244 | 0.00875 | 0 | 0.00361 | n/s | n/s |
|  |  | e=10 | 0.0356 | 0.00875 | 0 | 0.00648 | n/s | n/s |
|  |  | e=6 | 0.0442 | 0.016 | 0.00085 | 0.0176 | n/s | n/s |
|  | 10+ | perfect | 0.0097 | 0.00768 | 0 | 0.00078 | n/s | n/s |
|  |  | e=10 | 0.0141 | 0.00875 | 0 | 0.00127 | n/s | n/s |
|  |  | e=6 | 0.00926 | 0.00875 | 0 | 0.00172 | n/s | n/s |
| 2 (AC) | 2 | perfect | 8.21 | 7.56 | 6.99 | 7.04 | 0.00155 | n/s |
|  |  | e=10 | 8.18 | 7.56 | 6.98 | 7.02 | 0.00128 | n/s |
|  |  | e=6 | 7.5 | 6.96 | 6.45 | 6.48 | 0.00451 | n/s |
|  | 3 to 5 | perfect | 0.589 | 0.525 | 0.473 | 0.408 | n/s | n/s |
|  |  | e=10 | 0.573 | 0.524 | 0.473 | 0.406 | n/s | n/s |
|  |  | e=6 | 1.1 | 0.98 | 0.87 | 0.802 | 0.00445 | n/s |
|  | 6+ | perfect | 0.00662 | 0.001 | 0.0117 | 0.00104 | n/s | n/s |
|  |  | e=10 | 0.0222 | 0.001 | 0.0117 | 0.00158 | n/s | n/s |
|  |  | e=6 | 0.0358 | 0.0166 | 0.0117 | 0.00998 | n/s | n/s |
|  | 10+ | perfect | 0.00662 | 0.001 | 0 | 0 | 0.00038 | n/a |
|  |  | e=10 | 0.00662 | 0.001 | 0 | 0.00049 | n/s | n/s |
|  |  | e=6 | 0.00662 | 0.001 | 0 | 0.00391 | n/s | n/s |

### Table S17: continued

| **Repeat type** | | | **Mean repeat frequency by ORF type** | | | | **P value** | |
| --- | --- | --- | --- | --- | --- | --- | --- | --- |
| Motif  length | Copy  number | Mismatch  type | Hot | 1 removed  from hot | 2 removed  from hot | Non-hot | 1 removed  v non-hot | 2 removed  v non-hot |
| 2 (AG) | 2 | perfect | 9.2 | 8.97 | 8.66 | 8.79 | n/s | n/s |
|  |  | e=10 | 9.15 | 8.96 | 8.63 | 8.77 | n/s | n/s |
|  |  | e=6 | 8.31 | 8.11 | 7.8 | 8.02 | n/s | n/s |
|  | 3 to 5 | perfect | 0.695 | 0.735 | 0.613 | 0.705 | n/s | n/s |
|  |  | e=10 | 0.694 | 0.731 | 0.612 | 0.7 | n/s | n/s |
|  |  | e=6 | 1.26 | 1.38 | 1.21 | 1.28 | n/s | n/s |
|  | 6+ | perfect | 0.00954 | 0 | 0 | 0.00065 | n/s | n/s |
|  |  | e=10 | 0.00954 | 0 | 0 | 0.00167 | n/s | n/s |
|  |  | e=6 | 0.0285 | 0.0108 | 0.00567 | 0.0124 | n/s | n/s |
|  | 10+ | perfect | 0 | 0 | 0 | 0.00065 | n/s | n/s |
|  |  | e=10 | 0 | 0 | 0 | 0.00074 | n/s | n/s |
|  |  | e=6 | 0 | 0 | 0 | 0.00074 | n/s | n/s |
| 2 (CG) | 2 | perfect | 2.11 | 1.54 | 1.29 | 1.25 | 0.00072 | n/s |
|  |  | e=10 | 2.1 | 1.54 | 1.29 | 1.24 | 0.00076 | n/s |
|  |  | e=6 | 2.01 | 1.48 | 1.27 | 1.2 | 0.00211 | n/s |
|  | 3 to 5 | perfect | 0.106 | 0.0404 | 0.0476 | 0.0349 | n/s | n/s |
|  |  | e=10 | 0.106 | 0.0404 | 0.0476 | 0.0349 | n/s | n/s |
|  |  | e=6 | 0.161 | 0.0784 | 0.0887 | 0.067 | n/s | n/s |
|  | 6+ | perfect | 0 | 0 | 0 | 0 | n/a | n/a |
|  |  | e=10 | 0 | 0 | 0 | 0 | n/a | n/a |
|  |  | e=6 | 0 | 0 | 0 | 0.00017 | n/s | n/s |
|  | 10+ | perfect | 0 | 0 | 0 | 0 | n/a | n/a |
|  |  | e=10 | 0 | 0 | 0 | 0 | n/a | n/a |
|  |  | e=6 | 0 | 0 | 0 | 0 | n/a | n/a |
| 2 (all  motifs) | 2 | perfect | 24 | 23.7 | 22.8 | 23 | n/s | n/s |
|  |  | e=10 | 23.9 | 23.7 | 22.8 | 22.9 | n/s | n/s |
|  |  | e=6 | 21.9 | 21.7 | 20.9 | 21.1 | n/s | n/s |
|  | 3 to 5 | perfect | 1.82 | 1.79 | 1.72 | 1.74 | n/s | n/s |
|  |  | e=10 | 1.78 | 1.77 | 1.7 | 1.71 | n/s | n/s |
|  |  | e=6 | 3.18 | 3.2 | 3.15 | 3.13 | n/s | n/s |
|  | 6+ | perfect | 0.0405 | 0.00976 | 0.0117 | 0.0053 | n/s | n/s |
|  |  | e=10 | 0.072 | 0.0228 | 0.0291 | 0.0227 | n/s | n/s |
|  |  | e=6 | 0.109 | 0.0434 | 0.0182 | 0.0401 | n/s | n/s |
|  | 10+ | perfect | 0.0163 | 0.00868 | 0 | 0.00144 | 0.00512 | n/s |
|  |  | e=10 | 0.0207 | 0.00976 | 0 | 0.0025 | 0.00601 | n/s |
|  |  | e=6 | 0.0159 | 0.00976 | 0 | 0.00637 | n/s | n/s |
| 3 (all  motifs) | 2 | perfect | 13.9 | 13.5 | 13.9 | 13.6 | n/s | n/s |
|  |  | e=10 | 13.8 | 13.5 | 13.8 | 13.6 | n/s | n/s |
|  |  | e=6 | 12.4 | 12.2 | 12.5 | 12.3 | n/s | n/s |
|  | 3 to 5 | perfect | 0.895 | 0.897 | 0.726 | 0.66 | 0.00029 | n/s |
|  |  | e=10 | 0.844 | 0.839 | 0.678 | 0.636 | 0.00036 | n/s |
|  |  | e=6 | 2.03 | 1.99 | 1.82 | 1.8 | n/s | n/s |

### Table S17: continued

| **Repeat type** | | | **Mean repeat frequency by ORF type** | | | | **P value** | |
| --- | --- | --- | --- | --- | --- | --- | --- | --- |
| Motif  length | Copy  number | Mismatch  type | Hot | 1 removed  from hot | 2 removed  from hot | Non-hot | 1 removed  v non-hot | 2 removed  v non-hot |
| 3 (all  motifs) | 6+ | perfect | 0.0241 | 0.0358 | 0.0313 | 0.0159 | 0.00012 | n/s |
|  |  | e=10 | 0.0572 | 0.0683 | 0.0692 | 0.031 | < 0.0001 | n/s |
|  |  | e=6 | 0.0773 | 0.094 | 0.0955 | 0.0485 | < 0.0001 | n/s |
|  | 10+ | perfect | 0 | 0.0103 | 0.00284 | 0.00217 | n/s | n/s |
|  |  | e=10 | 0 | 0.022 | 0.00438 | 0.00479 | < 0.0001 | n/s |
|  |  | e=6 | 0 | 0.0285 | 0.00782 | 0.00898 | < 0.0001 | n/s |
| 4 (all  motifs) | 2 | perfect | 2.52 | 2.62 | 2.69 | 2.76 | n/s | n/s |
|  |  | e=10 | 2.42 | 2.56 | 2.64 | 2.69 | n/s | n/s |
|  |  | e=6 | 2.15 | 2.28 | 2.28 | 2.36 | n/s | n/s |
|  | 3 to 5 | perfect | 0.0196 | 0.0114 | 0.0213 | 0.0146 | n/s | n/s |
|  |  | e=10 | 0.1023 | 0.0456 | 0.0671 | 0.0722 | n/s | n/s |
|  |  | e=6 | 0.127 | 0.085 | 0.144 | 0.104 | n/s | n/s |
|  | 6+ | perfect | 0 | 0 | 0 | 0.00034 | n/s | n/s |
|  |  | e=10 | 0 | 0 | 0 | 0.00034 | n/s | n/s |
|  |  | e=6 | 0 | 0 | 0 | 0.00062 | n/s | n/s |
|  | 10+ | perfect | 0 | 0 | 0 | 0 | n/a | n/a |
|  |  | e=10 | 0 | 0 | 0 | 0 | n/a | n/a |
|  |  | e=6 | 0 | 0 | 0 | 0 | n/a | n/a |
| 5 (all  motifs) | 2 | perfect | 0.766 | 0.835 | 0.868 | 0.86 | n/s | n/s |
|  |  | e=10 | 0.755 | 0.817 | 0.84 | 0.83 | n/s | n/s |
|  |  | e=6 | 0.646 | 0.684 | 0.743 | 0.708 | n/s | n/s |
|  | 3 to 5 | perfect | 0 | 0.017 | 0.00924 | 0.00675 | n/s | n/s |
|  |  | e=10 | 0.00467 | 0.0249 | 0.0211 | 0.0251 | n/s | n/s |
|  |  | e=6 | 0.00219 | 0.0249 | 0.0247 | 0.0209 | n/s | n/s |
|  | 6+ | perfect | 0 | 0 | 0 | 0 | n/a | n/a |
|  |  | e=10 | 0 | 0 | 0 | 0.00083 | n/s | n/s |
|  |  | e=6 | 0 | 0 | 0 | 0.00093 | n/s | n/s |
|  | 10+ | perfect | 0 | 0 | 0 | 0 | n/a | n/a |
|  |  | e=10 | 0 | 0 | 0 | 0 | n/a | n/a |
|  |  | e=6 | 0 | 0 | 0 | 0.00053 | n/s | n/s |
| 6 (all  motifs) | 2 | perfect | 0.618 | 0.536 | 0.514 | 0.501 | n/s | n/s |
|  |  | e=10 | 0.575 | 0.501 | 0.492 | 0.472 | n/s | n/s |
|  |  | e=6 | 0.48 | 0.437 | 0.437 | 0.406 | n/s | n/s |
|  | 3 to 5 | perfect | 0.0495 | 0.0346 | 0.0157 | 0.0254 | n/s | n/s |
|  |  | e=10 | 0.0627 | 0.0336 | 0.0294 | 0.0287 | n/s | n/s |
|  |  | e=6 | 0.0616 | 0.0259 | 0.0177 | 0.0202 | n/s | n/s |
|  | 6+ | perfect | 0 | 0.00166 | 0 | 0.00013 | n/s | n/s |
|  |  | e=10 | 0.00839 | 0.00487 | 0 | 0.00326 | n/s | n/s |
|  |  | e=6 | 0.0135 | 0.00487 | 0 | 0.00074 | 0.00595 | n/s |
|  | 10+ | perfect | 0 | 0 | 0 | 0 | n/a | n/a |
|  |  | e=10 | 0.00662 | 0 | 0 | < 0.0001 | n/s | n/s |
|  |  | e=6 | 0.00662 | 0.00166 | 0 | 0.00031 | n/s | n/s |

1. Gerton JL, DeRisi J, Shroff R, Lichten M, Brown PO, Petes TD**: Inaugural article: global mapping of meiotic recombination hotspots and coldspots in the yeast Saccharomyces cerevisi**ae*. Proc Natl Acad Sci U S* A 2000**,** 97(21):11383-11390.
